# LC/MS/MS and GC/MS/MS metabolic profiling of *Leontodon hispidulus*, in vitro and in silico anticancer activity evaluation targeting hexokinase 2 enzyme

**DOI:** 10.1038/s41598-024-57288-4

**Published:** 2024-03-22

**Authors:** Noha Mokhtar Abd-El-Aziz, Mohamed Saeed Hifnawy, Rehab Ahmed Lotfy, Inas Youssef Younis

**Affiliations:** 1 Department of Medicinal and Aromatic Plants, Desert Research Center, Cairo, Egypt; 2 Department of Pharmacognosy, Faculty of Pharmacy, Cairo University, El Kaser El-Aini, Cairo, 11562 Egypt

**Keywords:** *Leontodon hispidulus *Boiss., Prostate carcinoma, LC-qToF-MS, GC–MS/MS, Hexokinase-2 enzyme, Pharmacology, Screening, Cancer therapy

## Abstract

*Leontodon hispidulus *Boiss is a wild annual plant growing in Egypt. The present study aims for the first time, to evaluate the phytochemical profile of the main secondary metabolites of the optimized ethanolic extract of the plant using Quadrupole Time-of-Flight Liquid chromatography-mass spectrometry and Gas chromatography-mass spectrometry. It also aims to assess the anticancer activity of its different fractions against the prostate carcinoma cell line. Moreover, an in-silico docking study was performed using the Hexokinase-two enzyme. LC-*q*ToF-MS analysis revealed the tentative identification of 36 phenolic compounds including the glycosides of (luteolin, quercetin, kaempferol, apigenin, isorhamnetin, and daidzein), coumarines (esculin, esculetin, and daphnetin), and phenolic acids (chlorogenic, caffeic, quinic, *P*-coumaric, and rosmarinic). GC–MS/MS analysis revealed the presence of 18 compounds where palmitic acid, myristic acid, alpha-amyrin, and beta-amyrin were the major ones. The cytotoxic activity results revealed that methylene chloride and ethyl acetate fractions showed the highest cytotoxic activity against the PC3 cell line, with IC_50_ values of 19, and 19.6 μg/ml, respectively. Interestingly, the docking study demonstrated that apigenin-7-*O*-glucoside, luteolin-7-*O*-glucoside, kaempferol-3-*O*-glucuronide, quercetin-4′-*O*-glucoside, esculin, rosmarinic acid, chlorogenic acid, and α-amyrin exhibited high affinity to the selected target, HEK-2 enzyme.

## Introduction

*Leontodon* is a rare wild plant of the family Asteraceae. It includes about 50 species that are geographically distributed through the Mediterranean, European, and Asian countries^1^. *Leontodon hispidulus* Boiss. is a member of the genus *Leontodon* that grows as a wild plant in Egypt. It is an annual plant, stemless with a rigid taproot, simple glandular hairs, rosette, oblong-oblanceolate leaves, and yellow–orange flowers^1^. Family Asteraceae is well-known for its flowering plants with a wide range of traditional uses. For example, *Carduus* species have often been used as anti-hemorrhoidal and cardiotonic remedies in traditional medicine, and *Onopordum tauricum* as a remedy for liver disease. The flowers and roots from *Onopordum acanthium* were used as antipyretic and diuretic agents, and *Centaurea solstitalis* is used in folk medicine to treat stomach problems, abdominal pain, herpes infections, and the common cold. *Tanacetum parthenium,* also known as feverfew in folk medicine and medieval aspirin, has been used as a remedy for headaches, migraine, nausea, vomiting, stomachaches, rheumatism, and other inflammations. Another plant with practical uses is *Bidens pilosa*, also known as Spanish needles, which grows mostly in subtropical and tropical regions. It has been used as a remedy for liver problems and to lower blood pressure. In addition, *Carthamus tinctorius* (safflower) is a treatment for rheumatism and osteoporosis in Korean herbal medicine. *Cichorium intybus* (chicory) is used in traditional medicine as a remedy for inflammation and liver disorders. Tonics from *C. intybus* have also been used to treat enlarged spleen and fever in Indian Ayurveda medicine, and a decoction from the leaves was used as a cure for rheumatism and gout. Tea prepared from *Achillea aleppica* and *Achillea biebersteinii* was recommended for abdominal pain. The aerial parts from *Chrysophthalmum montanum* were boiled and applied to wounds and other injuries. *Matricaria aurea* was recommended in the diet twice a day for bronchitis, sore throat, and cough^2^. Sesquiterpenoids especially guaiane-type compounds are the unique chemotaxonomic markers of *Leontodon* in the flower heads of *L. autumnalis* and the roots of *L. hispidus*^3,4^. Hypocretenolide glycoside was first isolated from *L. hispidus*, which had a potent cytotoxic activity against CD34( +) bone-marrow stem cell malignancy^5^ and anti-inflammatory activity^6^. In addition, other secondary metabolites had been reported in *Leontodon* taxa like phenolic acids (chicoric, chlorogenic, caffeoyl tartaric, and 3,5-dicaffeoyl quinic acids) as well as luteolin and its glycosides^7,8^. Moreover, a novel oleanene triterpene was isolated from the chloroform extract of the aerial parts of *L. filii* which showed a fungicidal activity^9^. In our previous work^10^ we reported that this plant species contains certain classes of secondary metabolites like sterols, flavonoids, coumarins, tannins, and terpenes through phytochemical screening. The High-performance liquid chromatography analysis showed the presence of chlorogenic acid, *p*-coumaric acid, rutin, quercetin, and kaempferol in *L. hispidulus* aerial parts 95% ethanolic extract. We also managed to optimize the plant extraction process of both the yield and total phenolic content by applying the Box-Behnken Design. The optimized extract produced the maximized concentration of phenolics (104.18 ± 0.37 μg/mg) with a minimum percentage of errors (− 0.27%) and flavonoid content (29.73 ± 0.0 μg/mg) using 201 ml of 74.5% ethanol/water at 72 h of extraction. The optimized extract showed good biological activities. The antioxidant activity using 2,2′-azino-bis (3-ethylbenzothiazoline-6-sulfonic acid) (ABTS) was found to be 41.89 mg Trolox-equivalent/gm, with 80% free radicals inhibition. Moreover, 100 mg/kg of the extract inhibited the edema in rats by 83.5% after 4 h of carrageenan injection as compared to 81.7% inhibition by indomethacin. The extract also showed good anticancer activity on both prostate carcinoma (PC3) and cervical carcinoma (HELA) cell lines (IC_50_ = 16.5 and 23 µg/ml, respectively)^10^. More studies were required to unveil the types of secondary metabolites of *L. hispidulus*. Prostate carcinoma is the majorly recorded malignancy in men, leading to many complications and finally mortality. Its early detection could be achieved by screening for serum prostate-specific antigen. Despite the advanced research to allow early diagnosis, the onset of the disease is often asymptomatic, it develops slowly and spreads from the prostate to other parts of the body. The standard primary treatment includes radical prostatectomy, radiation, and androgen deprivation therapy^11^. Unfortunately, patients with an initial response to treatment will relapse within three years with androgen-independent prostate cancer (PC3) which is rapidly fatal^12^. Generally, cancer treatment includes chemotherapy, radiotherapy, surgery, the transformation of stem cells, photodynamics, and immunotherapy. Severe side effects result from such treatments as limited bioavailability, toxicity, nonspecificity, fast clearance, and metastasis^13^. There are also many undesirable side effects that cancer patients find difficult to tolerate such as nausea, vomiting, anemia, fatigue, hair loss, appetite changes, constipation, bleeding, and infection. Considering this, there is a global need to look for more selective active compounds of natural origin. Hexokinase-2 enzyme (HEK-2) is an important target that is over-expressed in most human cancers and has been proven to be a promising target for cancer therapy. Although HEK-2 has been a potential target for cancer treatment for many years, several challenges remain in the discovery and design of efficient and selective HEK-2 inhibitors. These challenges arise out of the structural properties of HEK-2, including the high polarity of the active site of the enzyme and the difficulty in specifically inhibiting the different isoenzymes^14^. Computational or in-silico docking studies are important to assess the drug-like properties of lead compounds to predict their biological activity in-vivo. These studies help in the fast prediction of relevant properties and important parameters that should be met by a compound for it to be considered as a potential drug candidate. This objective is achieved by the application of automated software which can be used for making various predictions. Drug uptake, its absorption, evacuation, and associated hazardous effects are important factors for consideration in drug design and should be known in the early stages of drug development. Several important physicochemical properties like molecular weight, polar surface area, molecular flexibility, etc. have to be taken into consideration in drug designing. Toxicological assessment is another important aspect of drug discovery which predicts the safety and adverse effects of a drug. Therefore, bioactivity scores of probable drug leads against various human receptors can be used to predict and evaluate the probability of them acting as potential drug candidates in-vivo^15^.

The study aims to identify the major active compounds in *L. hispidulus* optimized extract, including phenolic compounds and terpenes, evaluate the cytotoxic activity of its successive fractions against prostate carcinoma cell lines (PC3), and test the inhibitory effect of the main compounds against HEK-2 enzyme in-silico.

## Materials

### Plant material collection and identification

The aerial parts of *Leontodon hispidulis* Boiss. were collected in March 2022 at the flowering stage from the north coast of Egypt, it was kindly identified by Dr. Soad Abdallah Hassan, Professor of flowering plants, Botany Department, Faculty of Science, Ain Shams University, Cairo, Egypt. Voucher specimens of the plant was deposited in the herbarium of “Pharmacognosy department, Faculty of Pharmacy, Cairo University, Cairo, Egypt” with the code number (21-3-17). Plant collection and experimental protocol were achieved after permission from the “Desert Research Center, Cairo, Egypt”, and the “Ethical Committee of the Faculty of Pharmacy, Cairo University, Cairo, Egypt” (serial number of the protocol: MP 1841). Experimental research and field studies on plants (either cultivated or wild), including the collection of plant material, must comply with relevant institutional, national, and international guidelines and legislation.

### Chemicals

#### Solvents for extraction and fractionation

Ethanol, petroleum ether, methylene chloride, ethyl acetate, butanol (all HPLC grade) were purchased from Sigma-Aldrich, Germany.

#### LC-qTOF-MS of the total extract

Mobile phase (negative mode): 5 mM ammonium formate buffer (pH = 8) containing 1% methanol. All solvents were HPLC grade and were kindly provided and prepared by Proteomics Laboratory of Children Cancer Hospital 57357, Cairo, Egypt.

#### GC–MS/MS of the petroleum ether fraction

Solvent: petroleum ether (HPLC grade) was purchased from Sigma Aldrich Chemicals, Germany.

Mobile phase: Helium gas at a flow rate of 1.0 ml/min at a splitless mode was provided by Central Laboratories Network, National Research Center, Giza, Egypt.

Silylation: 1% TMCS (trimethylchlorosilane) was provided by the National Research Center, Giza, Egypt.

#### Cytotoxic activity

Cell lines: Human prostate carcinoma (PC3) cell lines were purchased from the National Cancer Institute, Cairo, Egypt.

Doxorubicin: as a standard anticancer drug, it was purchased from Sigma Aldrich Chemicals, Germany.

Sulfo-Rhodamine B stain (SRB): for staining of the surviving cells, it was purchased from Sigma Aldrich Chemicals, Germany.

Acetic acid and Tris–EDTA buffer: were purchased from Sigma Aldrich Chemicals, Germany.

## Methods

### Preparation of the plant material

The powder of the aerial parts was extracted by maceration at room temperature using 201 ml of 74.5% ethanol/water at 72 h for 20 g powder to obtain the optimized extract with the maximum phenolic content^10^. The solvent was evaporated using a Rotatory Evaporator "BuchiRR-300, USA" at 45°. The resulting residue was subjected to fractionation with solvents of increasing polarity (petroleum ether, methylene chloride, ethyl acetate, butanol, and water).

### LC-qTOF-MS of the total extract

The separation process was performed using a Shimadzu UPLC system (Kyoto, Japan) equipped with a binary solvent delivery system and an autosampler, coupled with ACQUITY UPLC BEH-C18 (150 × 2.1 mm, 1.7 um: Waters, USA). The column temperature was maintained at 25 °C and mass spectrometry was performed on a Triple TOF™ 5600 + system with a Duo-Spray™ source operating in the Negative Electron Spray Ionization mode (ESI) (AB SCIEX, CA, USA). The flow rate was kept at 0.3 ml/min. The mobile phase A (5 mM ammonium formate buffer (pH = 8) containing 1% methanol) and mobile phase B (100% acetonitrile) were applied according to the gradient elution program demonstrated in Table 1.

**Table 1:** Gradient elution program for LC-qTOF-MS analysis.

Time (min)	Flow (ml/min)	Mobile phase A	Mobile phase B
0	0.3	90	10
1	0.3	90	10
21	0.3	10	90
25	0.3	10	90
25.01	0.3	90	10
28	0.3	90	10

#### Sample preparation for LC–MS/MS analysis

The optimized 74.5% ethanol/water extract of *L. hispidulus* was used in the LC–MS/MS analysis^10^. One ml mobile phase working solution (MP-WS) [DI-Water: Methanol: Acetonitrile—50: 25: 25] was added to 50 mg sample, followed by vortex for 2 min, then ultra-sonication for 10 min and centrifuge for 10 min at 10,000 rpm. 20 µl stock (50/1000 µl) was diluted with 1000 µl reconstitution solvent. Finally, the injected concentration was 1 µg/µl. The injection volume was 10 µl and 10 µl MP-WS as a blank.

#### Data processing

Respect Negative (1573 records) was the database used^16^. MasterView Program was used for feature (peaks) extraction from the Total ion chromatogram (TIC) based on the following criteria; features should have Signal-to-Noise > 5 (Non-targeted analysis), features intensities of the sample-to-blank > 5.

The following optimized parameters were applied; ion spray voltage (5.5 kV), ion source heater (550 °C), curtain gas [35 psi—gas 1 (nebulizer gas, 55 psi), gas 2 (heater gas, 55 psi)], declustering potential (60 eV), collision energy (30 eV), collision energy spread (15 eV). The scan range was from 50 to 800 m*/z* with a 250 ms accumulation time. An automated calibration delivery system (CDS) was applied for the regulation of the MS and the MS/MS automatically.

### GC–MS/MS of the petroleum ether fraction

The GC–MS/MS system (Agilent Technologies) was equipped with a gas chromatograph (7890B) and mass spectrometer detector (5977A) at Central Laboratories Network, National Research Centre, Giza, Egypt. The GC was equipped with HP-5MS column (30 m × 0.25 mm internal diameter and 0.25 μm film thickness). The petroleum ether fraction was subjected to a silylation process using 1% trimethylchlorosilane (TMCS). Helium was the carrier gas (1.0 ml/min flow rate) in a splitless mode and the injection volume was (0.1 µl). The following temperature program was applied [80 °C for 2 min; rising at 5 °C/min to 300 °C and held for 10 min]. The injector and detector were held at 280 °C and 300 °C, respectively. Mass spectra were obtained by electron ionization (EI) at 70 eV; using a spectral range of *m/z* 25–550 and solvent delay of 3.7 min. Identification of different constituents was determined by comparing the spectrum fragmentation pattern with those stored in Wiley and NIST Mass Spectral Library data.

### In-vitro cytotoxic activity

Measurement of the potential cytotoxic activity was performed on prostate carcinoma cell lines (PC3), using the successive fractions of the optimized ethanolic extract of *L. hispidulus* [petroleum ether, methylene chloride, ethyl acetate, butanol, and aqueous fractions], while doxorubicin was used as a positive control. The procedures were according to the Sulforhodamine-B assay (SRB)^10^**.** Each value is the mean of three replicates. Obtained values were presented as mean ± SD. Significant differences between the values were calculated using SPSS software (V.22) using One-way ANOVA and Tukey’s test. The difference is considered significant at (*P* ˂ 0.05).

### In-silico docking study using hexokinase-2 enzyme

The molecular modeling studies were carried out using Molecular Operating Environment (MOE, 2019.0102) software. The X-ray crystallographic structure of Hexokinase-2 complexed with the reference compound [2-amido-6-benzenesulfonamide glucosamine derivative (PDB ID: 5HFU)] was downloaded from the protein data bank (https://www.rcsb.org/structure/5HFU). All the tested compounds were compared to the reference compound.

## Results and discussion

### LC-qTOF-MS of *L. hispidulus* total extract

**Figure 1:**
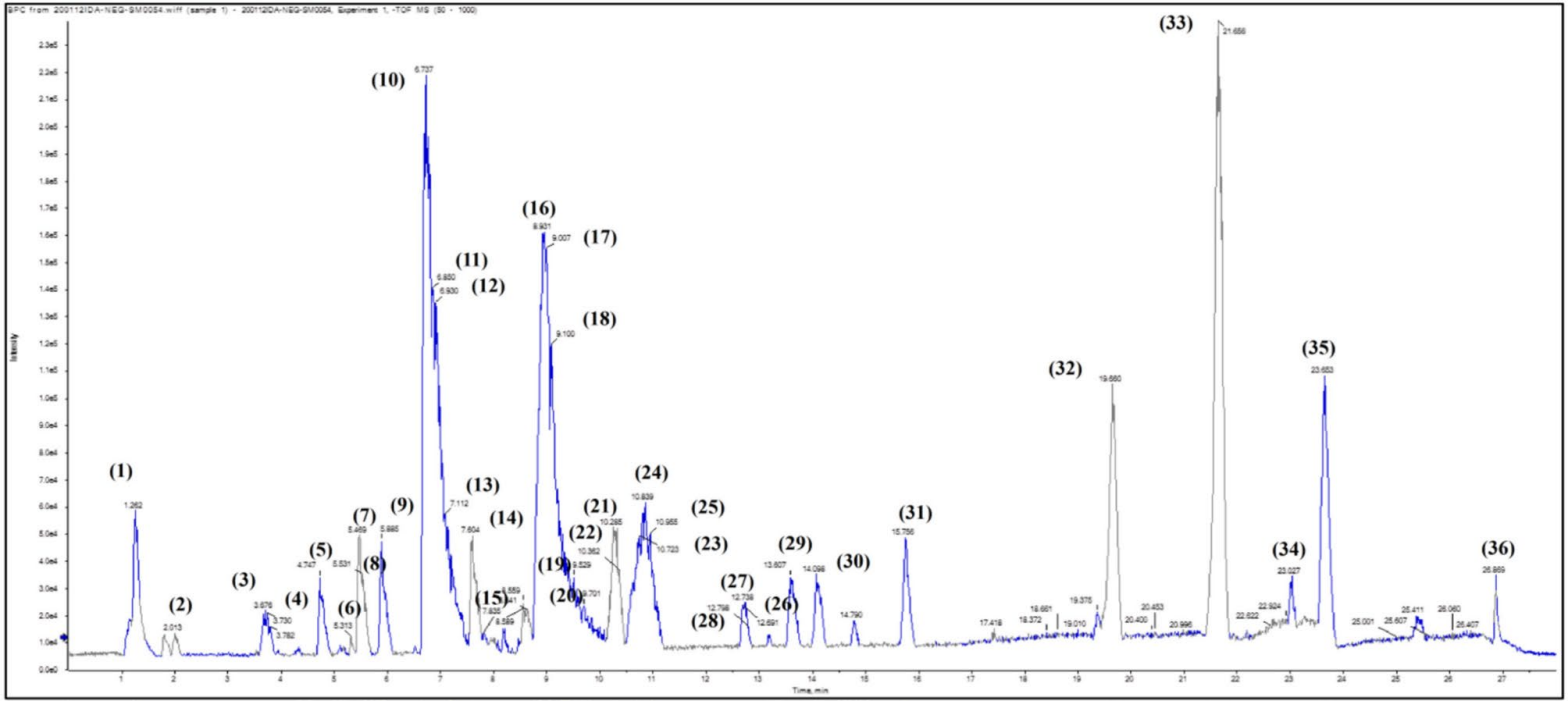
Base Peak Chromatogram of *Leontodon hispidulus* Boiss. Optimized extract in the Negative mode.

**Figure 2:**
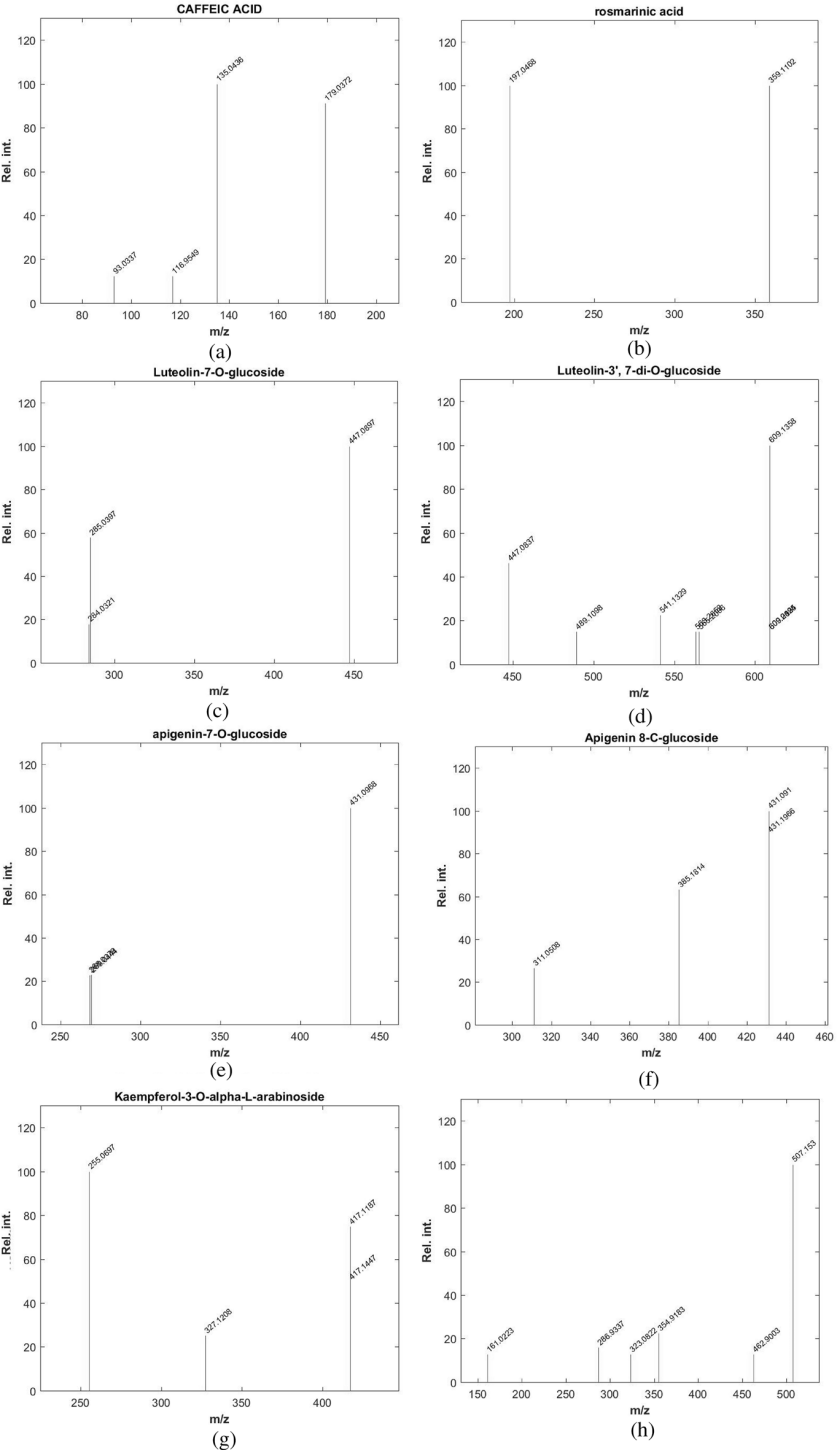

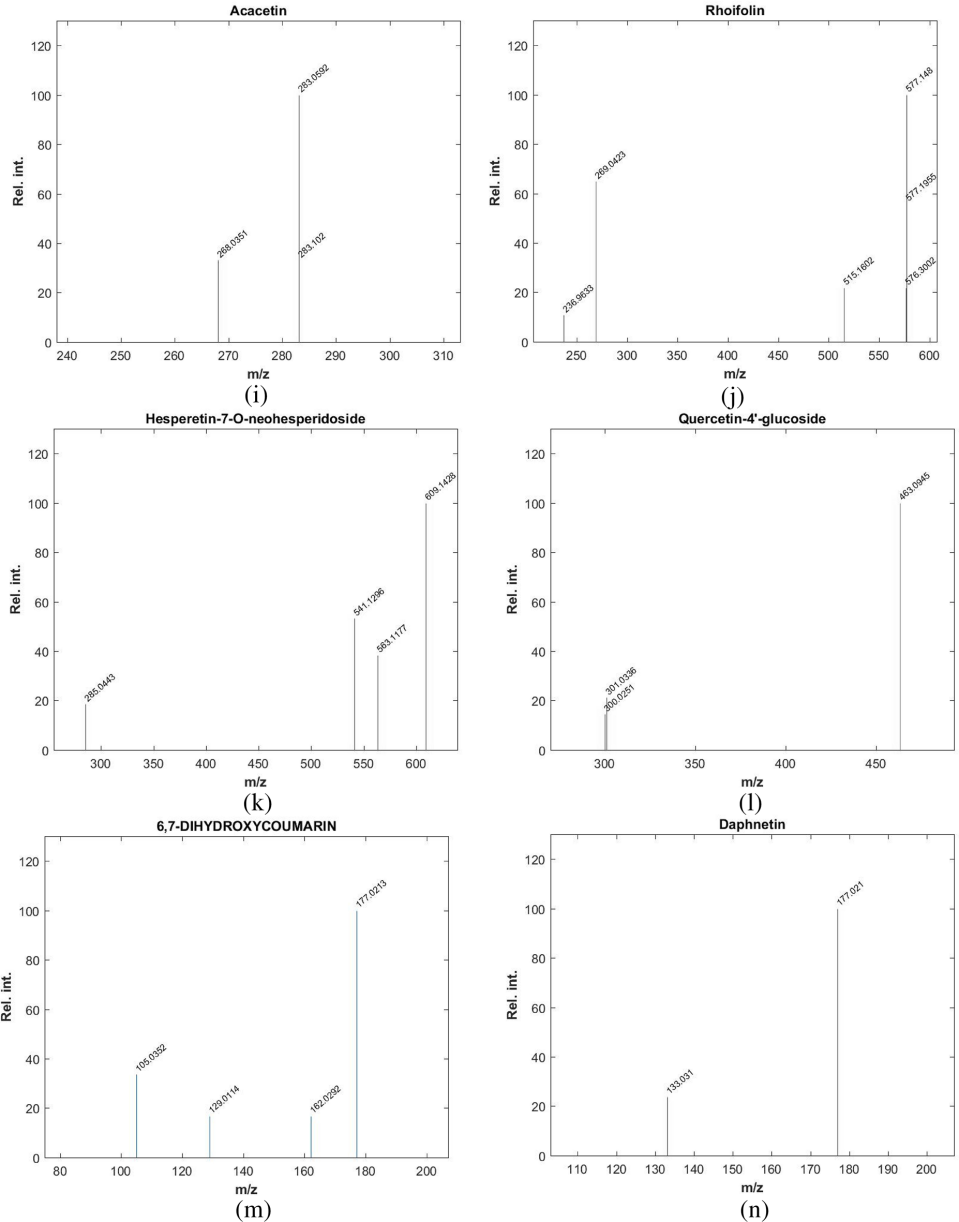
Fragmentation figures of the main identified compounds in *Leontodon hispidulus* Boiss. ethanolic extract (**a**) Ms/MS spectrum of caffeic acid, (**b**) Ms/Ms spectrum of rosmarinic acid, (**c**) Ms/Ms spectrum of luteolin-7-*O-*glucoside, (**d**) Ms/Ms spectrum of luteolin-3′,7-di-*O*-glucoside, (**e**) Ms/Ms spectrum of apigenin-7-*O*-glucoside, (**f**) MS/MS spectrum of apigenin 8-*C*-glucoside, (**g**) MS/MS spectrum of kaempferol-3-*O*-alpha-L-arabinoside, (**h**) MS/MS spectrum of syringetin-3-*O*-glucoside, (**i**) MS/MS spectrum of acacetin, (**j**) MS/MS spectrum of rhoifolin, (**k**) MS/MS spectrum of hesperetin-7-O-neohesperidoside, (**l**) MS/MS spectrum of quercetin-4′-glucoside, (**m**) MS/MS spectrum of 6,7-dihydroxy coumarin, (**n**) MS/MS spectrum of daphnetin.

Table 2 shows in detail the different identified phenolic compounds in *L. hispidulus* optimized ethanolic extract in the negative mode, while the base peak chromatogram is demonstrated in Fig. 1. Figure 2a–n demonstrates MS/MS spectrum figures of the major identified phenolic compounds.

**Table 2:** Tentative identification of the major secondary metabolites of *Leontodon hispidulus* Boiss. optimized extract in the negative mode.

Peak no.	Rt (min)	M-H *m/z*	Molecular formula	Error of *m/z*	Ms/Ms	Compound name
1	1.262	353	C_16_H_18_O_9_	0	191, 179, 173, 93, 85	Chlorogenic acid
2	2.013	191	C_7_H_12_O_6_	1	173, 135	Quinic acid
3	3.676	153	C_7_H_6_O_4_	0.5	109, 94	3,4-dihydroxybenzoic acid
4	3.730	163	C_9_H_8_O_3_	1.2	119, 91	*P*-coumaric acid
5	4.747	339	C_15_H_16_O_9_	-0.5	337, 293, 271, 194, 178, 177, 173, 149, 131	Esculin
6	5.313	137	C_7_H_6_O_3_	0.1	93	*P*-hydroxybenzoic acid
7	5.469	177	C_9_H_6_O_4_	-0.6	162, 133, 129, 105	6,7-dihydroxy coumarin (Esculetin)
8	5.531	179	C_9_H_8_O_4_	-0.8	135, 79	Caffeic acid
9	5.885	153	C_7_H_6_O_4_	0.5	136, 109, 67	2,5-dihydroxybenzoic acid
10	6.737	609	C_27_H_30_O_16_	-3	607, 565, 563, 541, 489, 447, 285	Luteolin-3ˋ,7-di-*O*-glucoside
11	6.850	177	C_9_H_6_O_4_	1.7	149, 133, 121, 105, 95, 89, 81	7,8-dihydroxy coumarin (Daphnetin)
12	6.930	359	C_18_H_16_O_8_	0.6	197	Rosmarinic acid
13	7.112	461	C_21_H_18_O_12_	-1	315, 285, 161, 132, 85	Kaempferol-3-Glucuronide
14	7.604	431	C_21_H_20_O_10_	-1.5	429, 363, 354, 295, 293, 285, 274, 179, 162, 158, 112	Kaempferol-3-*O*-L-rhamnoside
15	7.835	609	C_28_H_34_O_15_	1.4	565, 563, 541, 448, 355, 301, 285	Hesperetin-7-*O*-neohesperidoside
16	8.931	463	C_21_H_20_O_12_	-1.1	458, 301, 300, 274, 256	Quercetin-4ˋ-*O*-glucoside
17	9.007	431	C_21_H_20_O_10_	-1	341, 311, 269	Apigenin 8-*C*-glucoside (Vitexin)
18	9.100	167	C_8_H_8_O_4_	2	162, 152, 130, 124, 123, 108	5-Methoxysalicylic acid
19	9.529	623	C_28_H_32_O_16_	1.2	612, 577, 510, 477, 461, 357, 315, 314	Isorhamnetin-3-O- rutinoside
20	9.701	477	C_22_H_22_O_12_	-0.4	315, 300, 270, 180	Isorhamnetin-3-*O*- glucoside
21	10.265	577	C_27_H_30_O_14_	-1.2	576, 515, 269, 236	Rhoifolin
22	10.362	431	C_21_H_20_O_10_	1.1	269	Apigenin-7-*O*-glucoside
23	10.723	433	C_20_H_18_O_11_	-0.3	301, 300, 271, 255	Quercetin-3-D-xyloside
24	10.839	507	C_23_H_24_O_13_	0.6	463, 394, 345, 323, 286, 258, 179, 161, 153	Syringetin-3-*O*-glucoside
25	10.955	415	C_21_H_20_O_9_	-2.1	369, 304, 295, 253, 238, 192, 135	Daidzein-8-*C*-glucoside
26	12.691	417	C_20_H_18_O_10_	-1.6	327, 255	Kaempferol-3-*O*-alpha-L-arabinoside
27	12.738	593	C_30_H_26_O_13_	2.1	548, 528, 188	Kaempferol-3-*O*-(*p*-coumaroyl)-glucoside
28	12.798	445	C_21_H_18_O_11_	-0.9	377, 308, 269, 193, 161, 133	Baicalein-7-*O*-glucuronide
29	13.607	299	C_16_H_12_O_6_	0.1	284, 256, 227, 211, 135, 134, 107	3ˋ,5,7-trihydroxy-4ˋ-methoxyflavone (Diosmetin)
30	14.098	447	C_21_H_20_O_11_	0.6	285	Luteolin-7-*O*-glucoside
31	15.756	269	C_15_H_10_O_5_	1	225, 201, 181, 151, 117, 107	Apigenin
32	19.660	267	C_16_H_12_O_4_	0.5	266, 252, 239, 221, 183,131	Formononetin
33	21.656	301	C_15_H_10_O_7_	-1.3	301, 151, 107	Quercetin
34	23.027	285	C_15_H_10_O_6_	0.8	241, 201, 175, 151, 133, 121	Luteolin
35	23.653	283	C_16_H_12_O_5_	1	268, 240, 239, 212, 211, 151, 117, 107	Acacetin
36	26.869	271	C_15_H_12_O_5_	-0.8	151, 119	Naringenin

Peak no. Peak number, *Rt (min)* Retention time in minutes, *M–H (m/z)* Mass divided by Charge number in the Negative mode, *MS/MS* Mass fragmentation.

#### Identification of phenolic acids

Many phenolic acids have been identified in the negative mode of LC-qTOF-MS of *L. hispidulus* ethanolic extract. Chlorogenic acid was identified by the deprotonated molecule [M-H]^−^ at *m/z* = 353 and the fragment ion at *m/z* = 191 corresponding to the deprotonated quinic acid^17^. Quinic acid molecular ion (*m/z* = 191) was also detected^17^. The caffeic acid molecular ion was identified at *m/z* = 179 and its fragment ion *m/z* = 135 corresponding to (Caffeic acid—CO_2_)^18^. *P*-coumaric acid molecular ion appeared at *m/z* = 163 and its fragment ion (*m/z* = 119) corresponds to (quinic acid—CO_2_)^19^. Rosmarinic acid was identified by the molecular ion (*m/z* = 359), with its fragment ion (*m/z* = 197) which represents the loss of caffeic acid^20^. In the previous studies, few phenolic acids had been reported in *Leontodon* taxa (chicoric, chlorogenic, caffeoyl tartaric, and 3,5- dicaffeoyl quinic acids)^7^.

#### Identification of flavonoids and their glycosides

Different classes of flavonoids and their glycosides were identified in the LC-qTOF-MS spectrum of *L. hispidulus* optimized ethanolic extract including flavones, flavonols, isoflavones, and flavanones.

##### Flavones and their glycosides

Two flavones were detected, luteolin was tentatively assigned based on its parent ion at (*m/z* = 285) and by fragment ions (*m/z* = 151) for ring A and (*m/z* = 133) for ring B after ring C cleavage^21^. Similarly, apigenin at (*m/z* = 269) with the characteristic fragment ion (*m/z* = 117) for ring B as well as (*m/z* = 151) for ring A^21^. Two luteolin glycosides were identified. Luteolin-7-*O*-glycoside with its characteristic quasimolecular ion (*m/z* = 447) and fragment ion of the flavone aglycone *(m/z* = 285) with a diagnostic loss of hexose moiety (162 amu)^17,22^. The second one was luteolin -3ˋ,7- di-*O*-glycoside which was tentatively identified by its molecular ion (m/z = 609) as well as two characteristic fragment ions [(*m/z* = 447) after the loss of one hexose molecule and the distinct peak of the aglycone at (*m/z* = 285)]^23^. Two apigenin glycosides were tentatively identified. Apigenin-7-*O*-glycoside with molecular ion (*m/z* = 431) and fragment ion (*m/z* = 269) after loss of hexose molecule^22^. The other one was apigenin-8*-C*-glycoside (vitexin) with the same molecular ion and fragment ion of the aglycone, but with two characteristic fragment ions of the C-glycoside, (*m/z* = 341) indicating [M-H-90]^−^ and (*m/z* = 311) indicating [M-H-120]^−^^17^. Rhoifolin was tentatively identified with its molecular ion (*m/z* = 577) and the fragment ion (*m/z* = 269) corresponding to [M-H-Rham-Glc]^−^^24^. A methoxyflavone compound, 3′,5,7-trihydroxy-4′-methoxyflavone (diosmetin) was identified by the molecular ion (*m/z* = 299) and the characteristic fragments [M-H-CH_3_]^−^ with (*m/z* = 284), [M-H-CH_3_-CO]^−^ with (*m/z* = 256) and A-ring system with (*m/z* = 135)^25^. Acacetin (4′-*O*-methylated flavone) was identified at (*m/z* = 283) and the fragment ions (107, 117, and 151) resulted from Retro-Diels–Alder cleavage. Also, the fragment ions (239 and 211) were detected after the loss of CO_2_ and CO, respectively. Moreover, the fragment ions (268, 240, and 212) were detected due to the loss of CH_3_, CO, and CO groups, respectively^26^. Baicalein-7-*O*-glucuronide (trihydroxyflavone) was tentatively identified with the molecular ion (*m/z* = 445) and baicalein fragment ion (*m/z* = 269) corresponding to [M-H-176]^−^ due to loss of glucuronide moiety^27^. In the previous studies, luteolin and its glycosides were identified in *Leontodon* taxa^7,8^.

##### Flavonols and their glycosides

Quercetin (flavon-3-ol) was tentatively identified by its molecular ion (*m/z* = 301)^28^. Two quercetin glycosides were tentatively identified. The first one was quercetin-4ˋ-*O*-glycoside which was identified by its molecular ion (*m/z* = 463) and the fragment ion of the aglycone (*m/z* = 301) after loss of hexose molecule^29^. The other one was quercetin-3-D-xyloside and it was characterized by its molecular ion (*m/z* = 433) and the same peak of the aglycone (*m/z* = 301)^30^. Four kaempferol glycosides were tentatively identified. Kaempferol-3-Glucuronide was identified by its molecular ion (*m/z* = 461) and the characteristic loss of glucuronic acid moiety (176) leaving the aglycone peak at (*m/z* = 285)^28^. Similarly, kaempferol-3-O-alpha-L-arabinoside, kaempferol-3-*O*-(*p*-coumaroyl)-glucoside, and kaempferol-3-*O*-L-rhamnoside were tentatively identified using the online databases (RESPECT and MONA)^16^.

Two isorhamnetin (*O*-methoxy-flavonol) glycosides were also detected. Isorhamnetin-3-*O*-glucoside was identified by its molecular ion (*m/z* = 477), as well as the fragment ions of the aglycone (*m/z* = 315), [M-H-(Glc-H_2_O)-CH_3_]^−^ with (*m/z* = 300) and [M-H-(Glc-H_2_O)-CH_3_-Co]^−^ with (*m/z* = 270)^31^. The second one, isorhamnetin-3-*O*-rutinoside, was identified by its molecular ion (*m/z* = 623), the fragment ions [M-H-146]^−^ with (*m/z* = 477) and [M-H-162]^−^ with (*m/z* = 461) characteristic for the disaccharide moiety (rutinose: *m/z* = 308) and by the aglycone part (*m/z* = 315)^32,33^. Syringetin-3-*O*-glucoside (*O*-methylated-flavonol) was tentatively detected with the molecular ion (*m/z* = 507) and the aglycone (*m/z* = 345)^34^.

##### Isoflavones

Daidzein-8-*C*-glucoside was found with the molecular ion (*m/z* = 415), the fragment ion (*m/z* = 295) characteristic of C-glycoside [M-H-120]^−^ and the aglycone (*m/z* = 253) corresponding to [M-H-160]^−^^35^. Formononetin (4′-*O*-methyl isoflavone) was detected based on (*m/z* = 267) and the fragment ion (*m/z* = 252) due to the loss of the CH_3_ group^36^**.**

##### Flavanones

Naringenin molecular ion (*m/z* = 271) appeared with the fragment ions (*m/z* = 151) for ring A and (*m/z* = 119) for ring B^37^. Hesperetin-7-*O*-neohesperidoside was detected with the molecular ion (*m/z* = 609) and the fragment ion of the aglycone hesperetin (*m/z* = 301) indicating the loss of neohesperidose [M-308]^−^^38^.

#### Identification of Coumarins and their glycosides

For the first time, three coumarins have been tentatively identified in *L. hispidulus* ethanolic extract. Daphnetin (7,8-dihydroxy coumarin) was identified with its molecular ion (*m/z* = 177) and characteristic fragment ions (*m/z* = 149) corresponding to [M-H–CO]^−^, (*m/z* = 121) corresponding to [M-H-CO-CO]^−^ and (*m/z* = 133) corresponding to [M-H-CO_2_]^−^ which were formed due to the lactone ring structure^39^. Esculetin (6,7-dihydroxy coumarin) was found with its molecular ion (*m/z* = 177) and fragment ion (*m/z* = 133)^40^. Esculin was identified with the molecular ion (*m/z* = 339) and the fragment ion (*m/z* = 177) due to loss of sugar^40^.

### GC–MS/MS of the petroleum ether fraction of *L. hispidulus* extract

**Figure 3:**
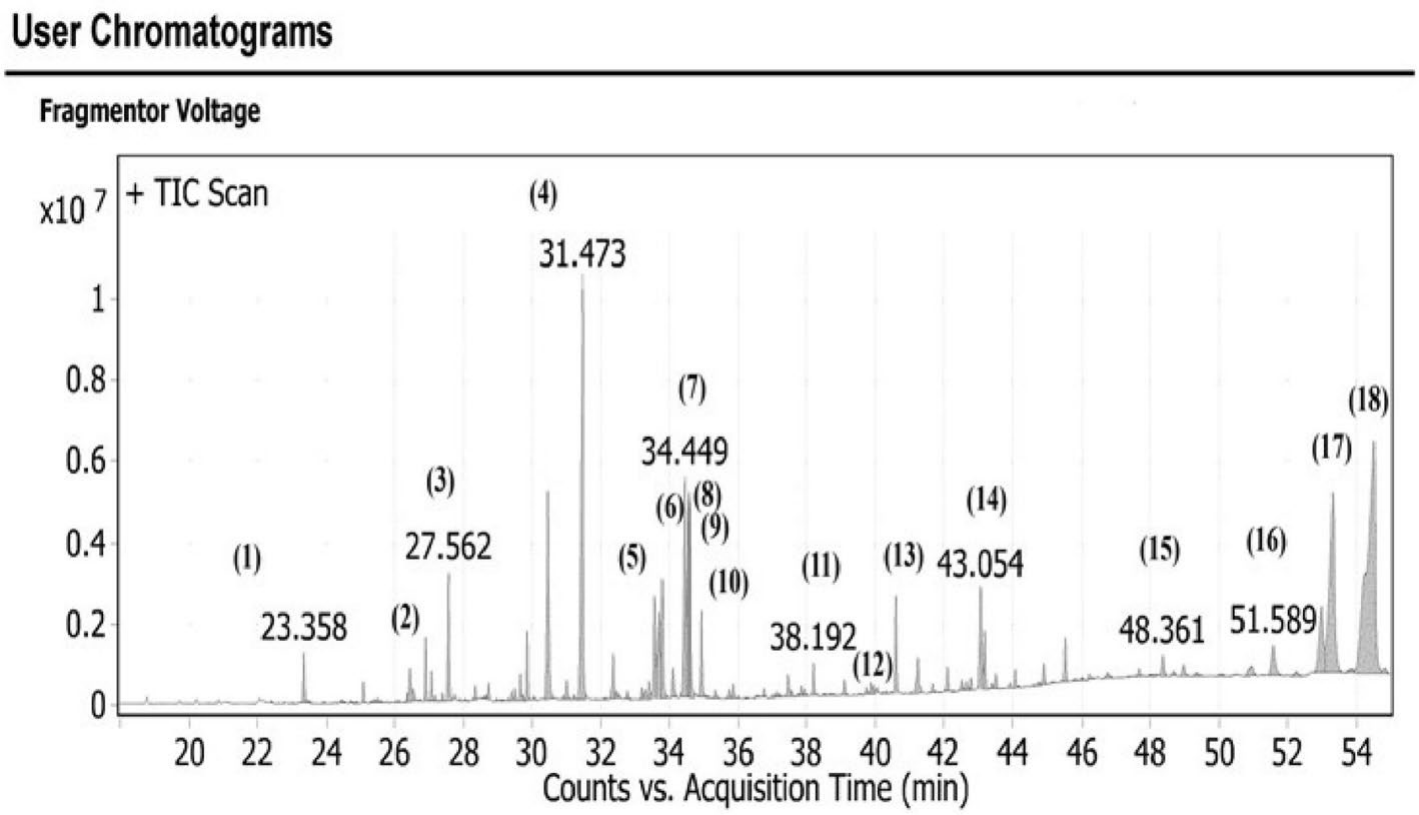
Chromatogram of the GC/MS of the petroleum ether fraction of *Leontodon hispidulus* Boiss.

**Figure 4:**
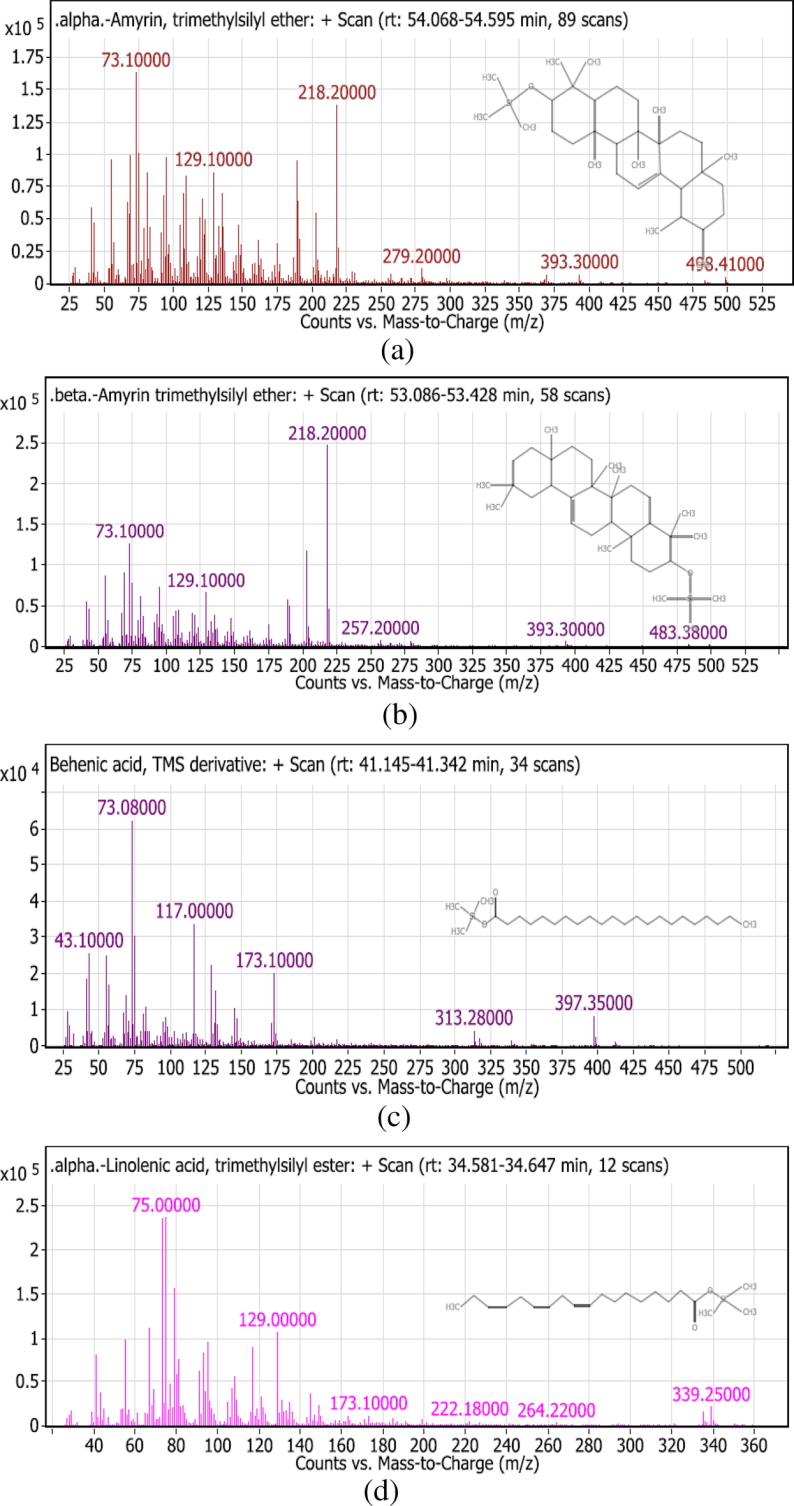

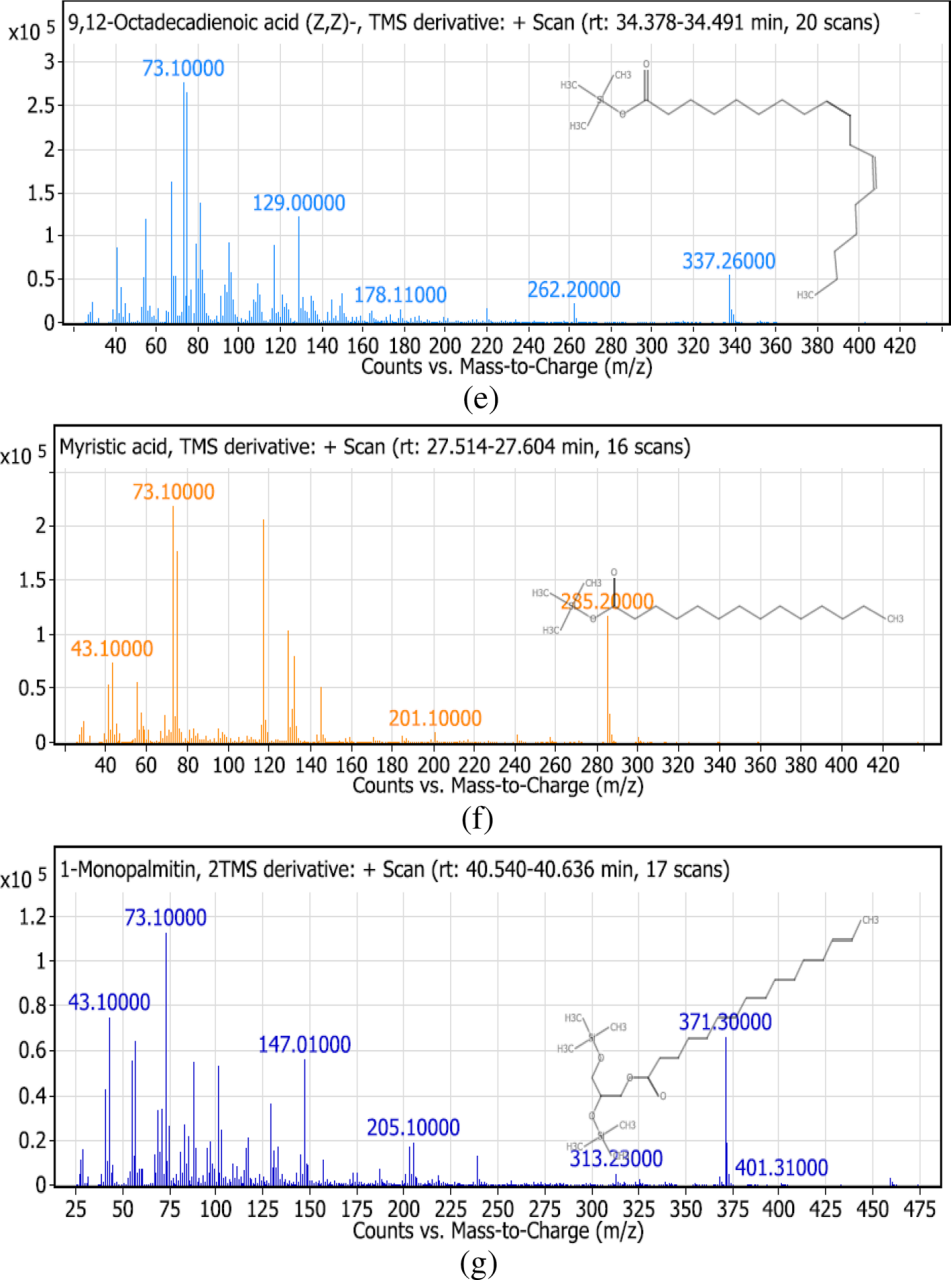
Fragmentation figures of the main identified compounds in *Leontodon hispidulus* Boiss. petroleum ether fraction, (**a**) Trimethylsilyl ether of Alpha-Amyrin, (**b**) Trimethylsilyl ether of Beta-Amyrin, (**c**) Trimethylsilyl derivative of Behenic acid, (**d**) Trimethylsilyl ester of Alpha-Linolenic acid, (**e**) Trimethylsilyl derivative of 9,12-Octadecadienoic acid, (**f**) Trimethylsilyl derivative of Myrestic acid, and (**g**) 2- trimethylsilyl derivative of 1-Monopalmitin.

Table 3 summarizes the identified compounds in *L. hispidulus* petroleum ether fraction by GC–MS/MS analysis. The total ion chromatogram is demonstrated in Fig. 3. Figure 4a–g demonstrates the MS/MS spectrum figures of the major identified compounds.

**Table 3:** The identified compounds in *Leontodon hispidulus* Boiss. petroleum ether fraction by GC–MS/MS analysis.

Peak no.	Rt (min)	Molecular weight	Molecular formula	Error	Ms/Ms	Compound name	Peak area %
1	23.316	272.49	C_15_H_32_O_2_Si	1.33	257, 201, 117, 73, 43	Dodecanoic acid, TMS derivative	1.10
2	26.436	256.42	C_16_H_32_O_2_	1	326, 211, 157, 88, 43	Tetradecanoic acid ethyl ester	1.82
3	27.562	300.55	C_17_H_36_O_2_Si	0.67	285, 201, 145, 132, 117, 73, 43	Myrestic acid, TMS derivative	7.25
4	31.473	328.6	C_19_H_40_O_2_Si	0.97	313, 269, 201, 145, 132, 117, 73, 43	Hexadecanoic acid TMS derivative	8.49
5	33.569	308.5	C_20_H_36_O_2_	1.03	339, 263, 67	Ethyl linoleate	1.72
6	33.791	368.7	C_23_H_48_OSi	1	213, 143, 73, 43	Silane,[(3,7,11,15- tetramethyl-2-hexadecenyl)oxy]trimethyl	0.77
7	34.449	352.6	C_21_H_40_O_2_Si	0.88	337, 262, 178, 145, 132, 129, 117,75,73	9,12-Octadecadienoic acid TMS derivative	7.08
8	34.563	354.64	C_21_H_42_O_2_Si	0.34	339, 264, 185, 117, 73	9-Octadecenoic acid TMS derivative	7.93
9	34.593	350.61	C_21_H_38_O_2_Si	1.42	335, 264, 222, 173, 145, 132, 129, 117, 75,73	alpha-linolenic acid trimethylsilyl derivative	9.02
10	34.94	356.65	C_21_H_44_O_2_Si	0.65	341, 297, 201, 73, 43	Octadecanoic acid trimethylsilyl ester	1.88
11	38.192	384.7	C_23_H_48_O_2_Si	0.15	369, 325, 201, 117, 73, 43	Eicosanoic acid trimethylsilyl ester	1.04
12	40.588	474.86	C_25_H_54_O_4_Si_2_	0.89	401, 371, 313, 205, 147, 73, 43	1-Monopalmitin 2TMS derivative	2.14
13	41.241	412.76	C_25_H_52_O_2_Si	1.61	397, 313, 173, 117, 73, 43	Behenic acid TMS derivative	1.82
14	43.054	500.9	C_27_H_56_O_4_Si_2_	1.21	483, 395, 263, 203, 129, 73	2-Oleoylglycerol 2TMS derivative	1.37
15	48.361	282.47	C_18_H_34_O_2_	0.45	484, 394, 255, 129, 83, 55	Oleic acid	2.26
16	51.589	486.88	C_32_H_58_OSi	1.03	486, 357, 203, 129, 55	Stigmast-5-ene, 3-beta (trimethylsiloxy) (24S)	2.12
17	53.314	498.9	C_33_H_58_OSi	0.35	483, 393, 257, 218, 216, 129, 73	beta-Amyrin trimethylsilyl derivative	20.13
18	54.512	498.9	C_33_H_58_OSi	0.37	498, 483, 393, 279, 218, 203, 189, 129, 73	alpha- Amyrin trimethylsilyl derivative	20.38

Peak no. Peak number, *Rt (min)* Retention time in minutes, *MS/MS* Mass fragmentation.

#### Identification of the major compounds in *L. hispidulus* petroleum ether fraction.

Trimethylsilyl derivatives of the fatty acids show a characteristic fragmentation pattern. Hexadecanoic acid TMS derivative (palmitic acid) is the major saturated fatty acid that exists. A characteristic peak appears at *m/z* = 313 [M-15] due to the loss of methyl group from the silyl part which makes the cation more stable. A characteristic peak appears at *m/z* = 145 [(CH_3_)_3_SiCO_2_C_2_H_4_]^+^ due to the cleavage of the β, γ bond (relative to the carbonyl group). Another characteristic peak appears at *m/z* = 132 [(CH_3_)_3_SiCO_2_CH_3_]^+^ due to Mclafferty rearrangement. Generally, in TMS derivatives, the peak m/z = 73 [CH_3_)_3_Si]^+^ is the base peak. Another important peak appears at *m/z* = 117 [(CH_3_)_3_SiCO_2_]^+^. A similar fragmentation pattern applies to other saturated TMS derivatives of fatty acids as myristic acid^41^. 9,12-Octadecadienoic acid TMS derivative is the main unsaturated fatty acid detected. The peak *m/z* = 337 [M-15] appears due to the loss of methyl group from the silyl part. The peak *m/z* = 73 [CH_3_)_3_Si]^+^ is the base peak. The compound follows a similar fragmentation pattern to the saturated fatty acids except that the peaks at *m/z* = 132 [(CH_3_)_3_SiCO_2_CH_3_]^+^ and at *m/z* = 145 [(CH_3_)_3_SiCO_2_C_2_H_4_]^+^ are less prominent^41^. Two triterpenes were detected for the first time in the genus *Leontodon*, α-amyrin (ursane-type) and β-amyrin (oleanane-type). Both follow the Retro Diels–Alder fragmentation pattern. Similar to the fatty acids, the base peak is also at *m/z* = 73 [CH_3_)_3_Si]^+^ for both α- and β-amyrin. Also, the peak at *m/z* = 483 [M-15] due to the loss of methyl group from the silyl part appears in both compounds. The peak at *m/z* = 218 is prominent in both which is characteristic of Retro Diels–Alder fragmentation. The peak at *m/z* = 189 is characteristic of α-amyrin, while the peak at *m/z* = 216 is indicative of β-amyrin^42^. Guaiane-type sesquiterpenoid compounds were discovered previously in the flower heads of *L. autumnalis*^3^ and the roots of *L. hispidus*^4^**.** Moreover, a hypocretenolide glycoside was isolated from *L. hispidus*, which had a potent cytotoxic activity^5^ and anti-inflammatory activity^6^.

### Cytotoxic activity of *L. hispidulus* successive fractions against Prostate carcinoma (PC3)

**Figure 5:**
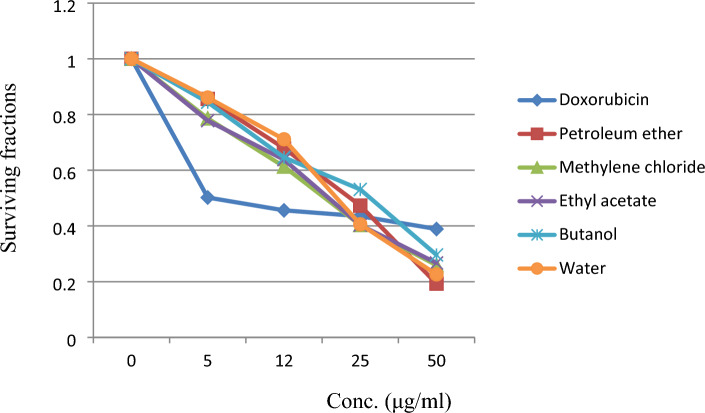
Cytotoxic activity of *Leontodon hispidulus* Boiss. successive fractions and doxorubicin against prostate carcinoma cell line.

**Figure 6:**
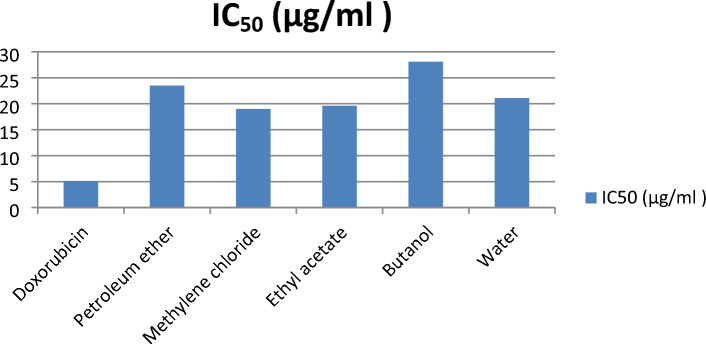
IC_50_ of *Leontodon hispidulus* Boiss. successive fractions and Doxorubicin against prostate carcinoma cell line.

Results of the cytotoxic study are demonstrated in Figs. 5 and 6, where IC_50_ values of the different fractions (petroleum ether, methylene chloride, ethyl acetate, butanol, and aqueous) against prostate carcinoma were (23.5, 19, 19.6, 28.1, and 21.1 μg/ml, respectively). The most active fractions were methylene chloride and ethyl acetate with very close IC_50_ values (19 and 19.6 μg/ml, respectively), while the standard doxorubicin IC_50_ was 5.1 μg/ml. In our previous work^10^, the IC_50_ of *L. hispidulus* total optimized ethanolic extract against the PC3 cell line was 16.5 μg/ml which was more potent anticancer activity than each fraction alone. This result indicates a synergistic activity between the five fractions. Statistical analysis of the data of the surviving fractions was performed by SPSS software (V.22) using One-way ANOVA and Tukey’s test. Table 4 represents The One-way ANOVA results for the surviving fractions of *Leontodon hispidulus* Boiss. comparing the successive fractions to doxorubicin. Table 5 demonstrates The Multiple Comparison: Post Hoc Test—Tukey’s HSD results. Interestingly, both methylene chloride and ethyl acetate fractions showed a non-significant difference from the standard doxorubicin through the lowest concentrations (05.00, 12.50, and 25.00 µg/ml), while the difference was significant (higher in activity than doxorubicin) at the highest concentration (50.00 µg/ml). As demonstrated in Table 5, group (2) [methylene chloride fraction] showed a non-significant difference from group (6) [doxorubicin] through the first three concentrations [05.00 µg/ml, *P*-value = 0.077, 12.50 µg/ml, *P*-value = 0.319] and [25.00 µg/ml, *P*-value = 0.976]. While the difference was significant (higher in activity than doxorubicin) at the highest concentration [50.00 µg/ml, *P*-value = 0.007*]. Group (3) [ethyl acetate fraction] showed a similar pattern, the difference from group (6) [doxorubicin] was non-significant through the first three concentrations [05.00 µg/ml, *P*-value = 0.088, 12.50 µg/ml, *P*-value = 0.193] and [25.00 µg/ml, *P*-value = 0.975]. While the difference was significant (higher in activity than doxorubicin) at the highest concentration [50.00 µg/ml, *P*-value = 0.011*]. This result came in agreement with the close values of the IC_50_ of methylene chloride fraction, ethyl acetate fraction, and doxorubicin (19, 19.6, and 5.1, respectively), hence, the high anticancer activity of both fractions against the PC3 cell line. Group (5) [aqueous] came in the third position in activity after groups (2 and 3). It showed a significant difference (higher activity) from group (6) [doxorubicin] at the first two lowest concentrations [05.00 µg/ml, *P*-value = 0.019*, 12.50 µg/ml, *P*-value = 0.038*] and at the highest concentration [50.00 µg/ml, *P*-value = 0.001*], hence, the IC_50_ = 21.1 μg/ml. Group (1) [petroleum ether] showed a significant difference from group (6) [doxorubicin] at both the lowest concentration [05.00 µg/ml, *P*-value = 0.021*] and the highest concentration [50.00 µg/ml, *P*-value = 0.000*]. Finally, group (4) [butanol] showed a significant difference from group (6) [doxorubicin] at the lowest concentration [05.00 µg/ml, *P*-value = 0.026*]. It is worthy to mention, that all the successive fractions (except for the butanol fraction) showed a significantly higher anticancer activity against the PC3 cell line than doxorubicin at the highest concentration (50.00 µg/ml). In agreement with a previous study, the anticancer activity of *L. hispidus* was mainly linked to the isolated compound (hypocretenolide glycoside)^5^. Similarly, the anticancer potential of *L. saxatilis*^43^ was attributed to the presence of cichoric acid and three flavone glycosides [apigenin 4′-*O*-β-D-glucoside, luteolin 7-*O*-β-D-glucoside, and luteolin 4′-*O*-β-D-glucoside] against plasma cell myeloma (OPM2) cell line. Ultimately, the results suggest that, the promising anticancer activity of *L. hispidulus* Boiss. is due to the synergistic action of compounds of different chemical groups (flavonoid glycosides, phenolic acids, coumarins, and terpenes).

**Table 4:** The One-way ANOVA results for the surviving fractions of *Leontodon hispidulus* Boiss. comparing the successive fractions to doxorubicin.

Conc. (µg/ml)	Surviving fractions	Sum of squares	Degree of freedom (df)	Mean square	F-value	Significance
05.00	Between groups	0.280	5	0.056	4.464	0.016*
	Within groups	0.151	12	0.013		
	Total	0.430	17			
12.50	Between groups	0.119	5	0.024	3.037	0.053
	Within groups	0.094	12	0.008		
	Total	0.212	17			
25.00	Between groups	0.040	5	0.008	2.746	0.070
	Within groups	0.035	12	0.003		
	Total	0.075	17			
50.00	Between groups	0.068	5	0.014	11.161	0.000*
	Within groups	0.015	12	0.001		
	Total	0.083	17			

*The mean difference is significant at (*P* < 0.05).

Groups: petroleum ether, methylene chloride, ethyl acetate, butanol, aqueous and doxorubicin.

**Table 5:** The Multiple Comparison: Post Hoc Test – Tukey’s HSD results for the surviving fractions of *Leontodon hispidulus* Boiss. comparing the successive fractions to doxorubicin.

Conc. (µg/ml)		Significance (*P*-value)			
		05.00	12.50	25.00	50.00
Group (I) versuss Group (J)					
1	2	0.967	0.934	0.619	0.261
	3	0.950	0.991	0.615	0.172
	4	1.000	0.997	0.771	0.033*
	5	1.000	0.997	0.658	0.849
	6	0.021*	0.079	0.947	0.000*
2	1	0.967	0.934	0.619	0.261
	3	1.000	0.999	1.000	1.000
	4	0.985	0.997	0.105	0.788
	5	0.956	0.747	1.000	0.849
	6	0.077	0.319	0.976	0.007*
3	1	0.950	0.991	0.615	0.172
	2	1.000	0.999	1.000	1.000
	4	0.974	1.000	0.104	0.908
	5	0.936	0.905	1.000	0.706
	6	0.088	0.193	0.975	0.011*
4	1	1.000	0.997	0.771	0.033*
	2	0.985	0.997	0.105	0.788
	3	0.974	1.000	0.104	0.908
	5	1.000	0.939	0.117	0.216
	6	0.026*	0.163	0.310	0.059
5	1	1.000	0.997	0.658	0.849
	2	0.956	0.747	1.000	0.849
	3	0.936	0.905	1.000	0.706
	4	1.000	0.939	0.117	0.216
	6	0.019*	0.038*	0.984	0.001*
6	1	0.021*	0.079	0.947	0.000*
	2	0.077	0.319	0.976	0.007*
	3	0.088	0.193	0.975	0.011*
	4	0.026*	0.163	0.310	0.059
	5	0.019*	0.038*	0.984	0.001*

*The mean difference is significant at (*P* < 0.05).

Standard Error for conc. 05.00 µg/ml = 0.09144.

Standard Error for conc. 12.50 µg/ml = 0.07214.

Standard Error for conc. 25.00 µg/ml = 0.04401.

Standard Error for conc. 50.00 µg/ml = 0.02854.

Group 1: Petroleum ether fraction, Group 2: Methylene chloride fraction, Group 3: Ethyl acetate fraction, Group 4: butanol fraction, Group 5: Aqueous fraction, Group 6: Standard doxorubicin.

### In-silico docking study using hexokinase-2 enzyme

**Figure 7:**
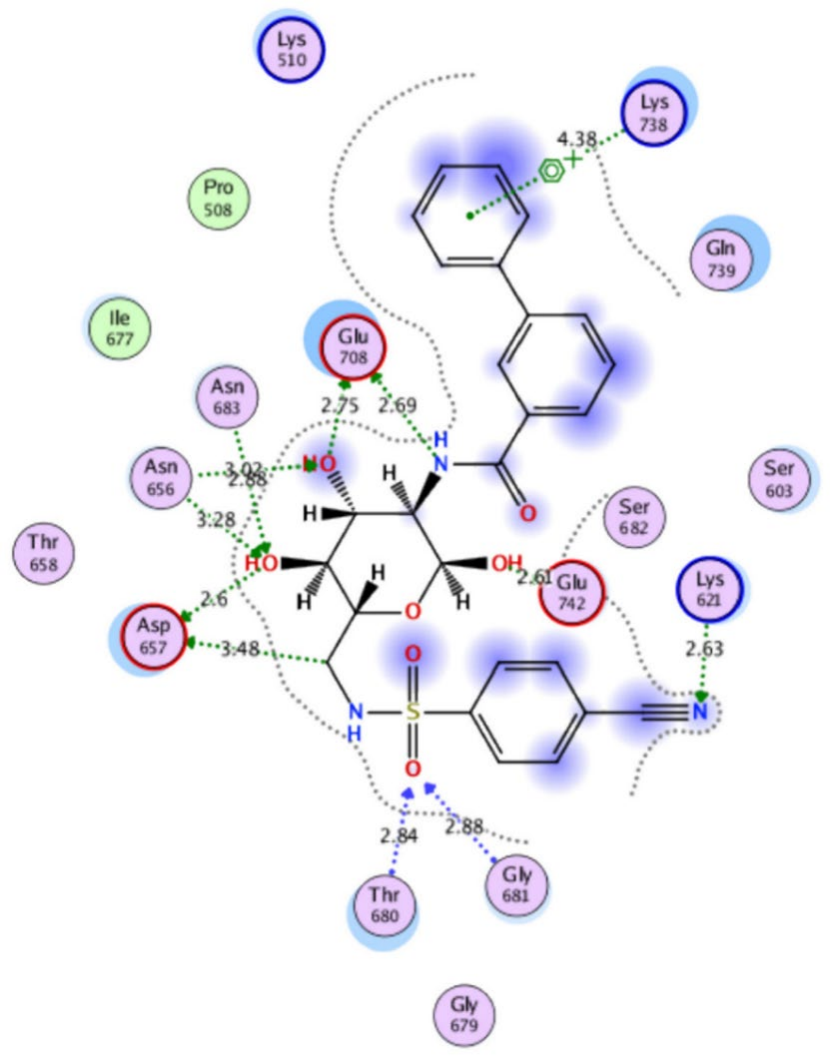
2D interactions of the native ligand within the hexokinase-2 active site.

**Figure 8:**
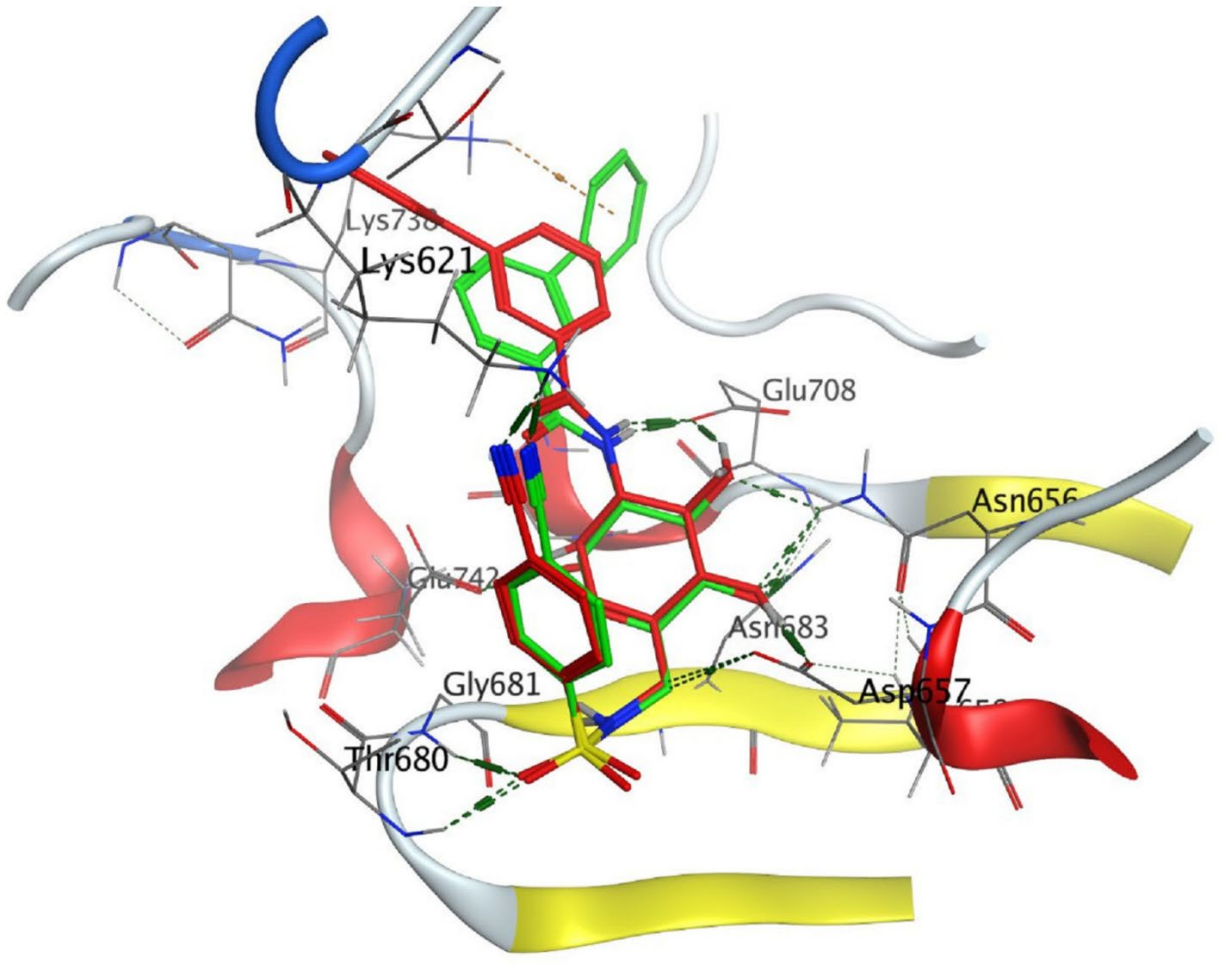
3D representation of the superimposition of the co-crystallized (green) and the docking pose (red) of the native ligand in the active site of hexokinase-2.

**Figure 9:**
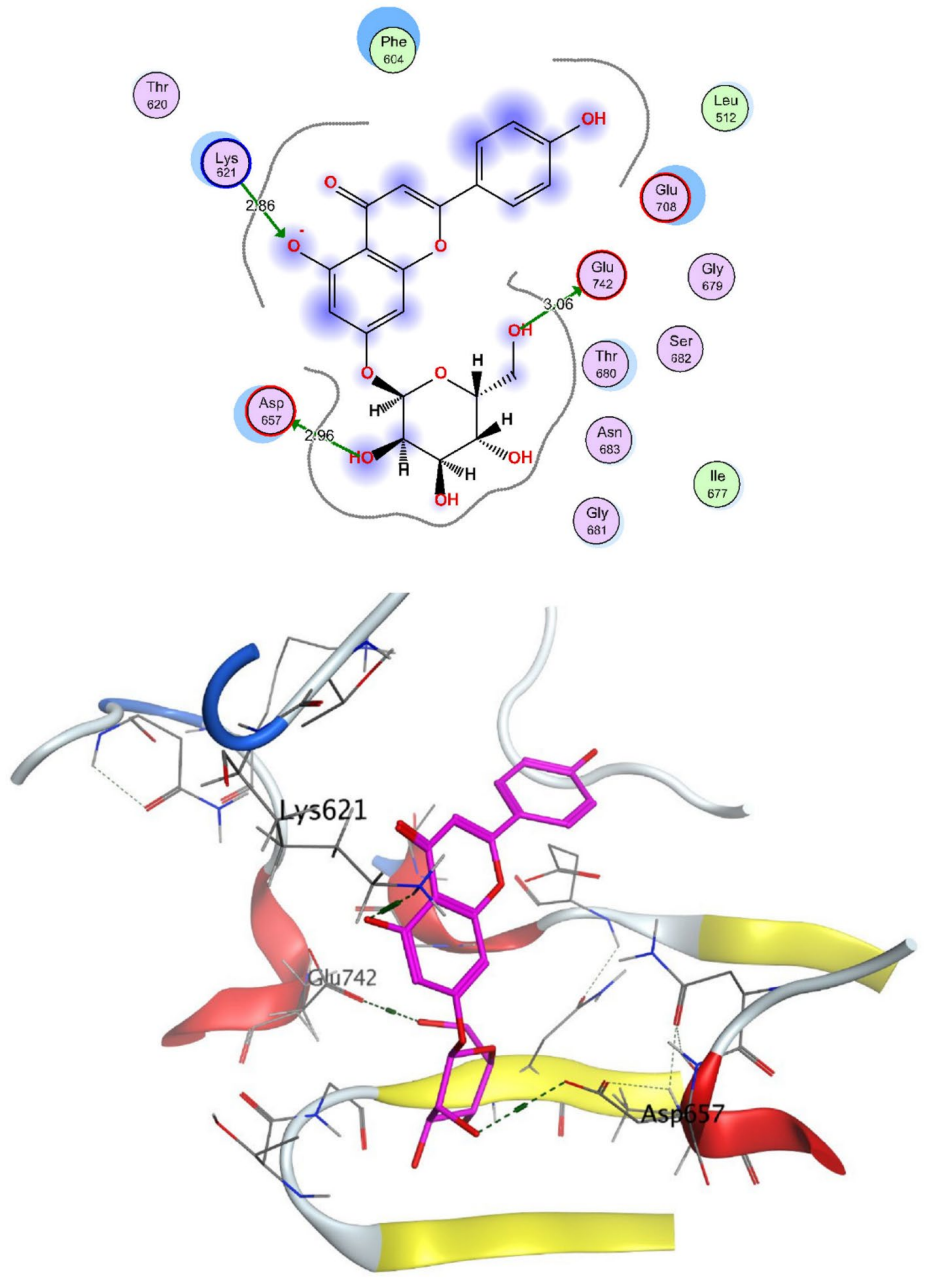
2D and 3D interactions of Apigenin-7-*O*-glucoside within hexokinase-2 active site.

**Figure 10:**
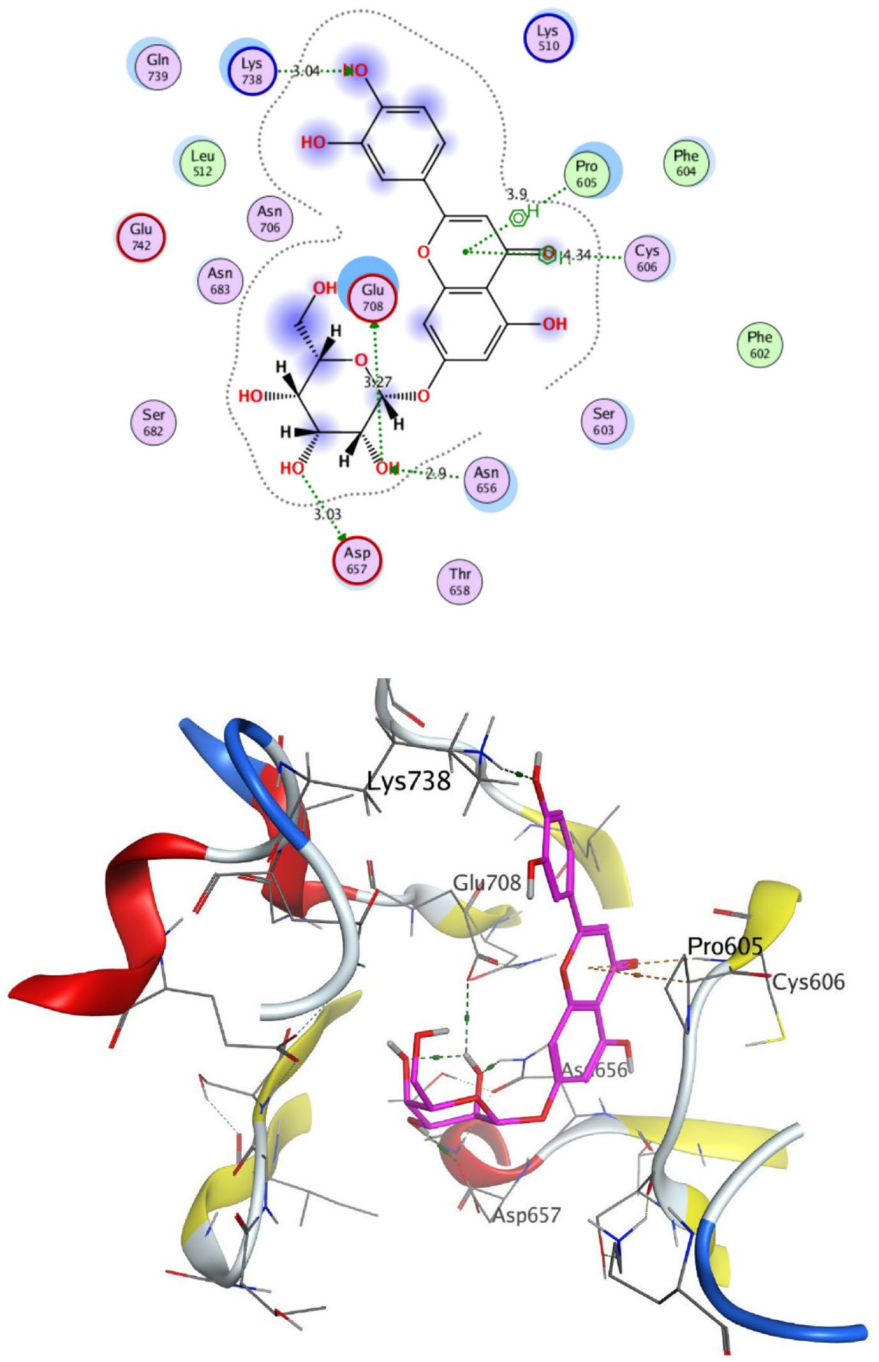
2D and 3D interactions of Luteolin-7-*O*-glucoside within hexokinase-2 active site.

**Figure 11:**
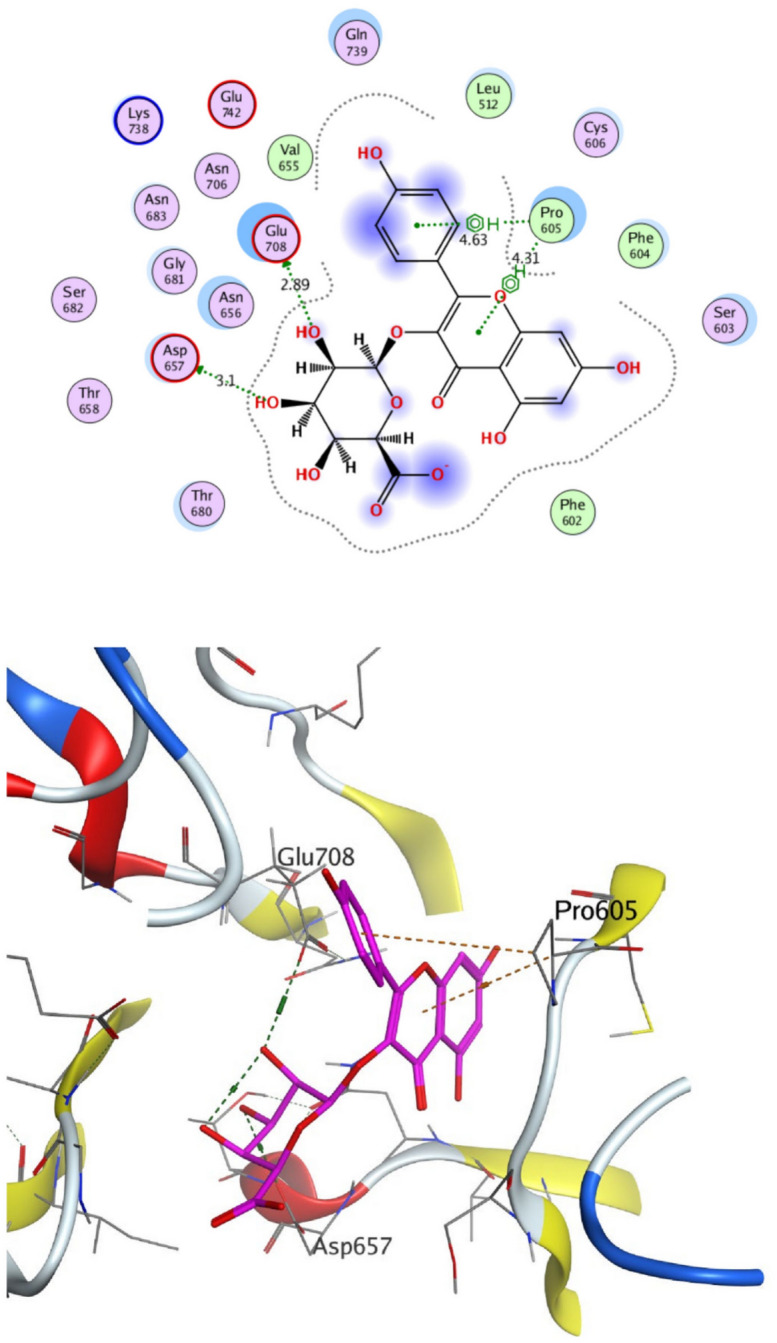
2D and 3D interactions of Kaempferol-3-*O*-glucuronide within hexokinase-2 active site.

**Figure 12:**
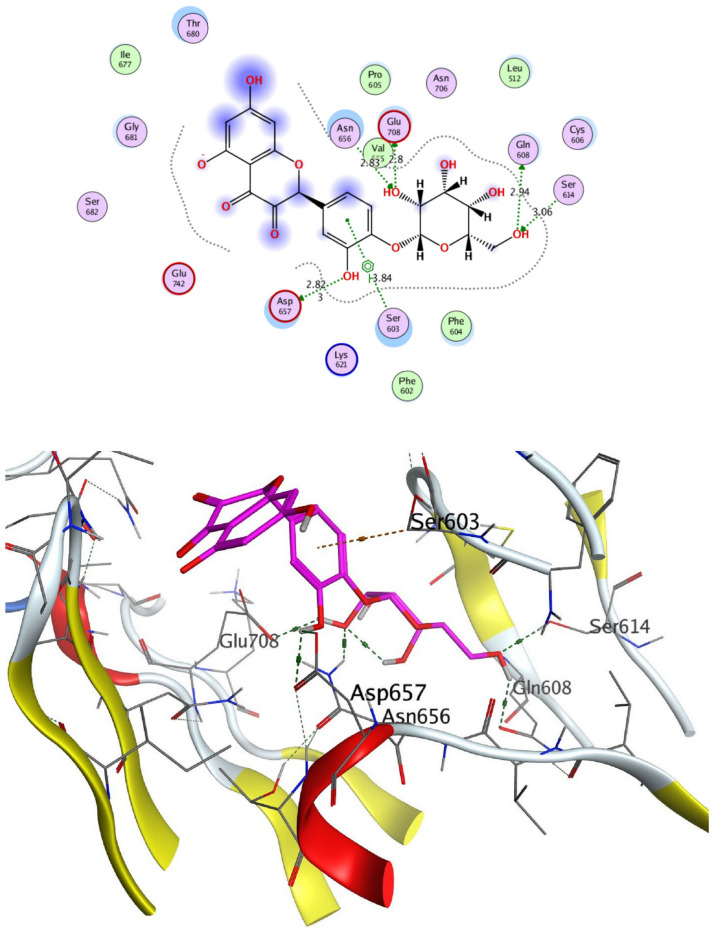
2D and 3D interactions of Quercetin-4ˋ-*O*-glucoside within hexokinase-2 active site.

**Figure 13:**
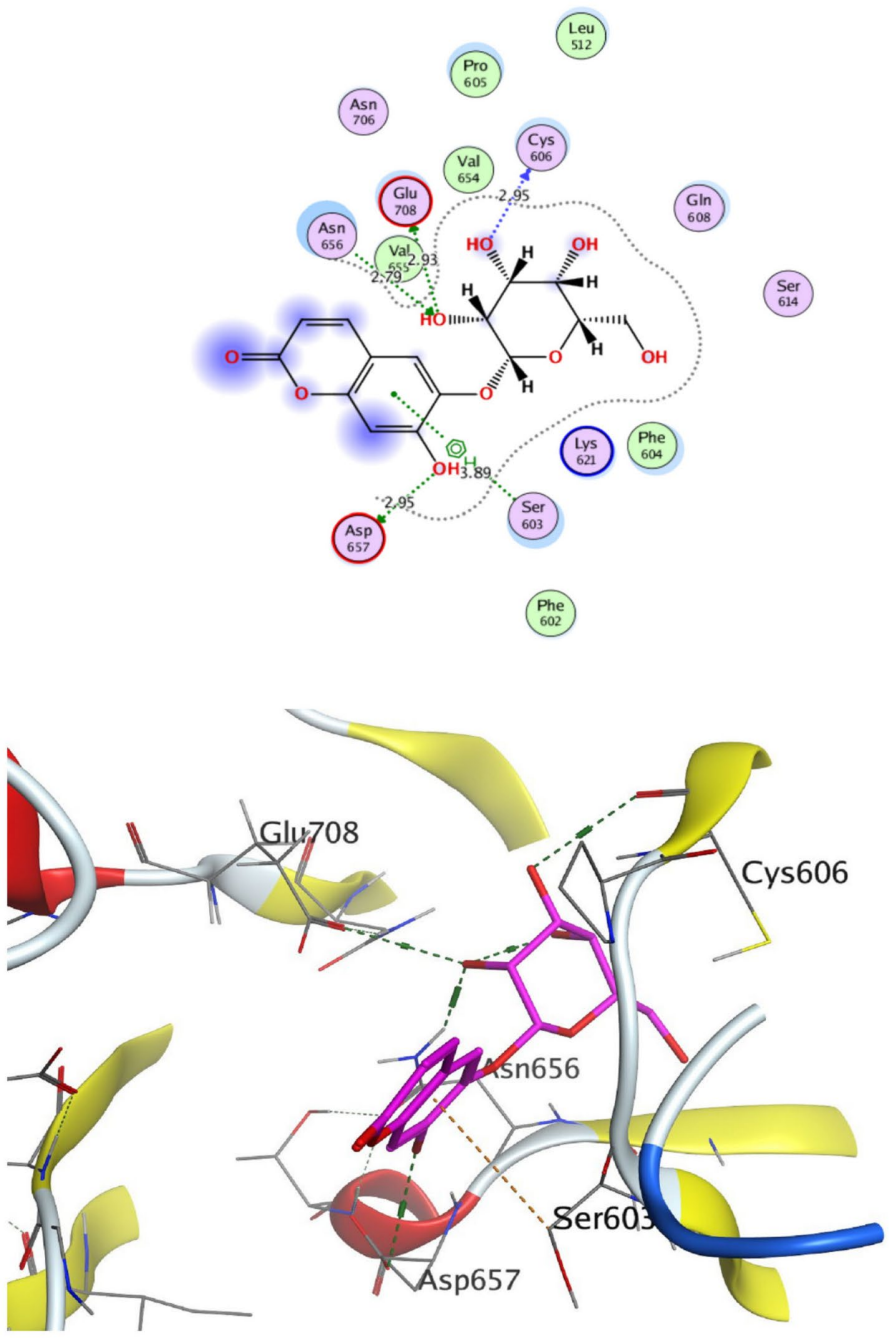
2D and 3D interactions of Esculin within hexokinase-2 active site.

**Figure 14:**
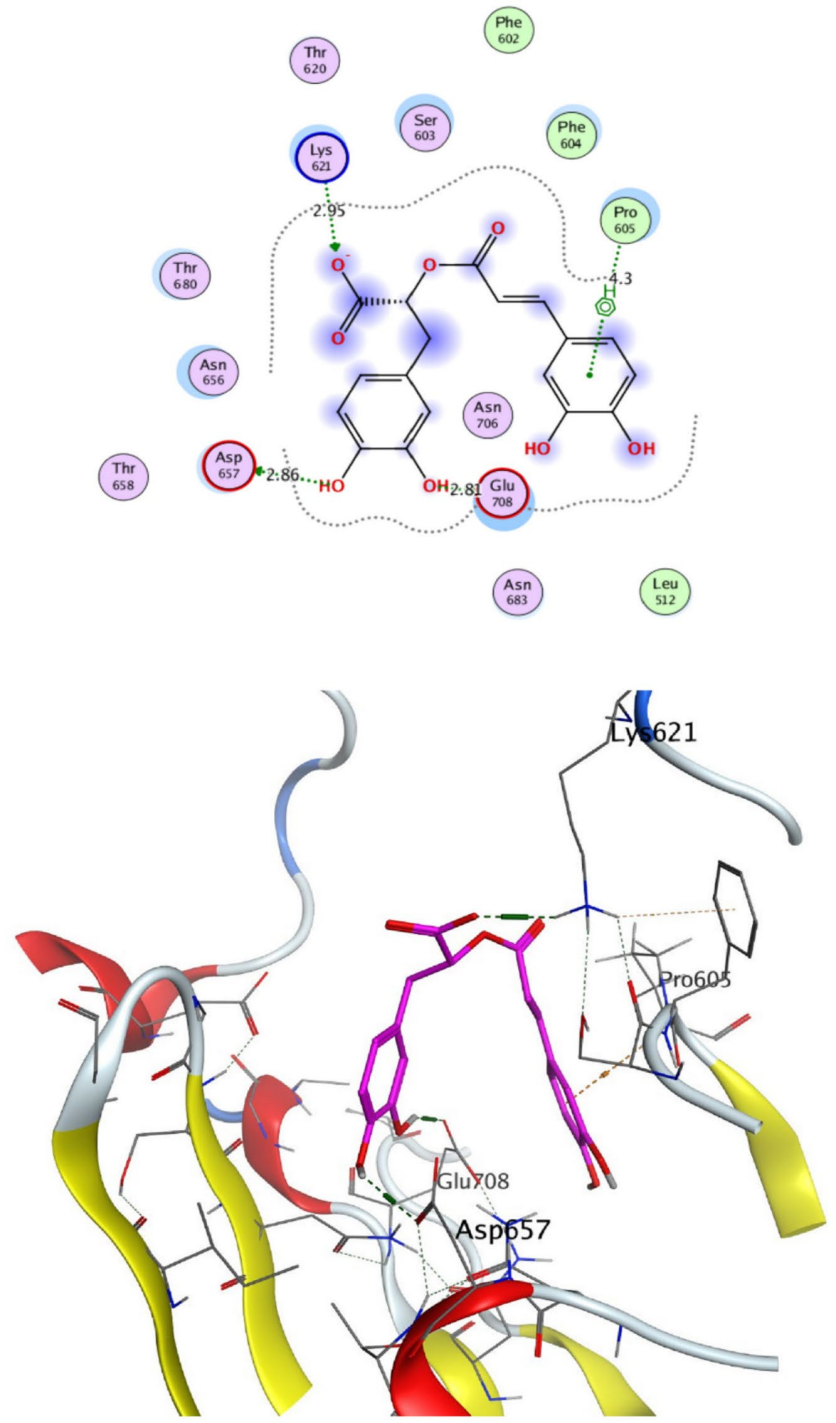
2D and 3D interactions of Rosmarinic acid within hexokinase-2 active site.

**Figure 15:**
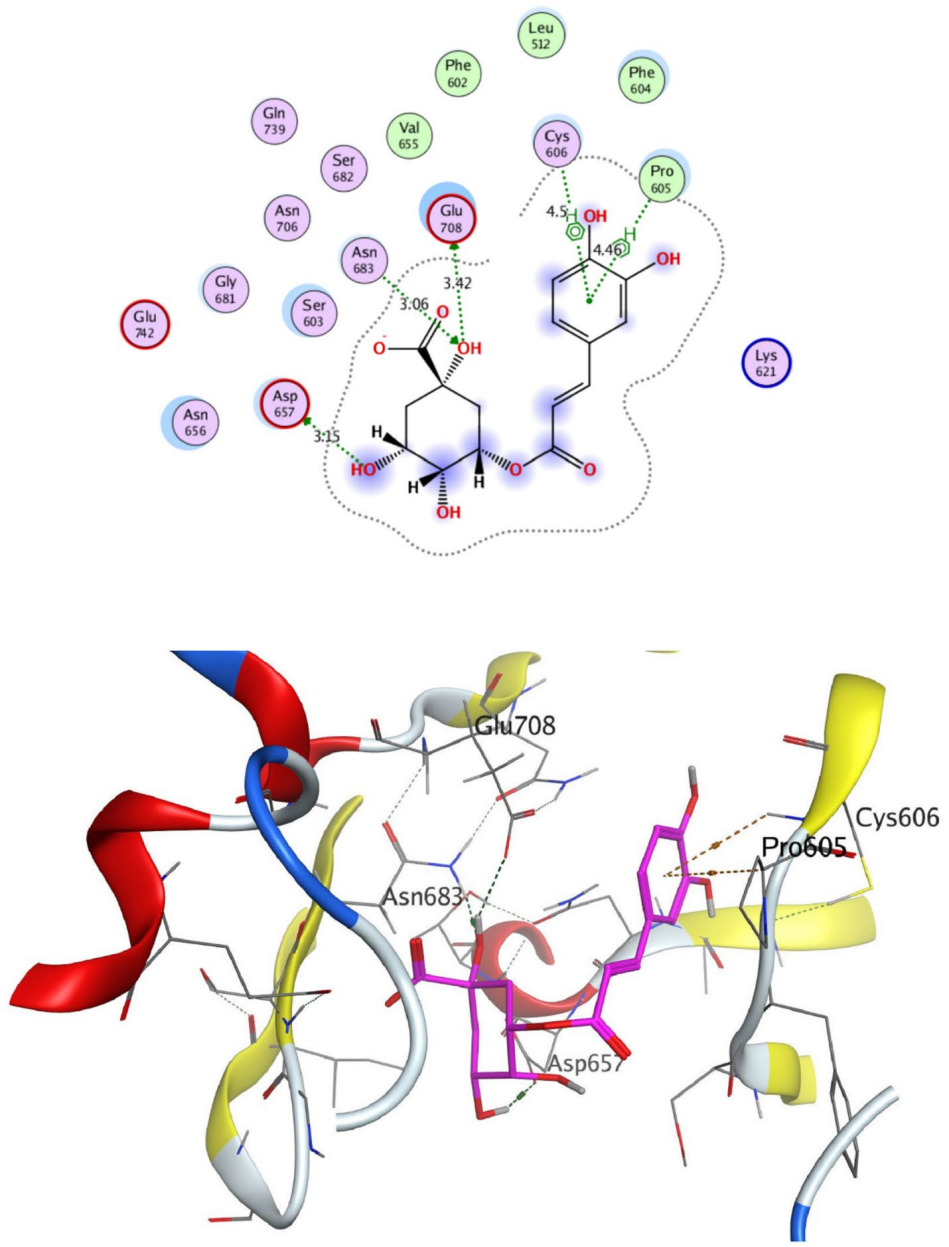
2D and 3D interactions of Chlorogenic acid within hexokinase-2 active site.

**Figure 16:**
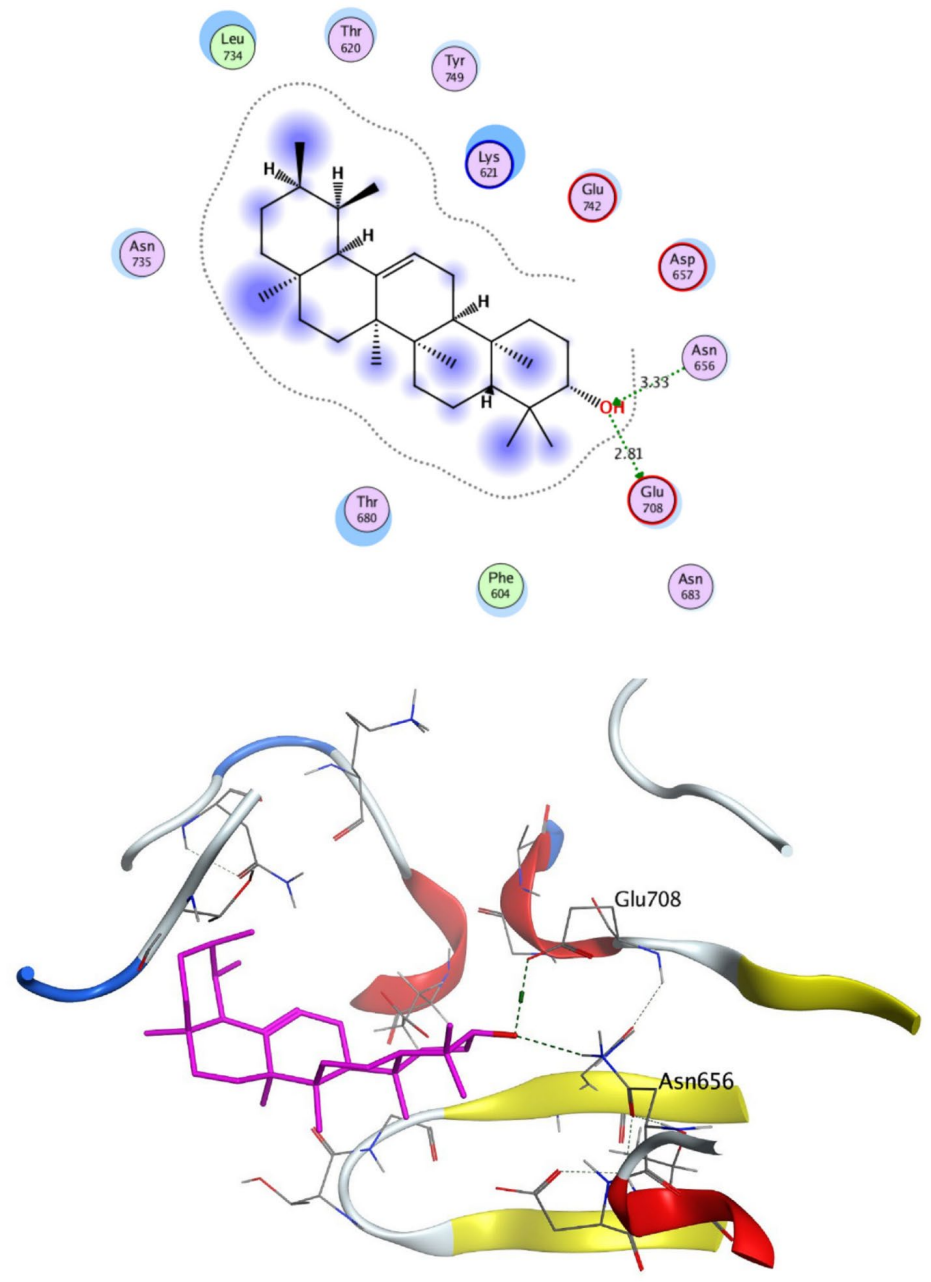
2D and 3D interactions of Alpha-Amyrin.

The structural properties of HEK-2 represent a challenge due to the high polarity of the active sites of the enzyme and the difficulty in specifically inhibiting the different isoenzymes^14^. Therefore, eight identified compounds of varying polarity were selected for the in-silico docking study. The molecular modeling study was carried out using Molecular Operating Environment (MOE, 2019.0102) software. The docking setup was first validated by self-docking of the co-crystallized ligand in the vicinity of the binding site of the enzyme, the docking score (S) was − 8.018 kcal/mol. and root mean square deviation (RMSD) was 1.931 Å Figs. 7 and 8. As demonstrated in (Table 6), all the tested eight compounds showed promising inhibitory effects on the HEK-2 enzyme. Apigenin-7-*O*-glucoside (Fig. 9) was the most active compound with a binding score of -7.412 through three hydrogen bonds with HEK-2. Two bonds as a H-bond donor with Asp657 and Glu742 amino acid residues and one bond as a H-bond acceptor with Lys621. luteolin-7-*O*-glucoside (Fig. 10) was found to be the second most active compound with a very close score of − 7.342. IT also formed three H-bonds with HEK-2, two as a H-bond donor with the amino acid residues Asp657 and Glu708, and one as a H-bond acceptor with Asn656. There were also two additional H–π interactions with Cys606 and Pro605 amino acid residues. Both compounds proved to possess an anticancer activity^44^**.** This result was in good agreement with the previous study of Palombo^45^ and a previous study on *L. saxatilis*^43^ that reported the anticancer activity of both apigenin 4′-*O*-β-D-glucoside, luteolin 7-*O*-β-D-glucoside**.** Kaempferol-3-O-glucuronide (Fig. 11) and quercetin-4′-O-glucoside (Fig. 12) were next in activity due to the similar structure to the first two compounds. Kaempferol-3-O-glucuronide showed anticancer activity using the Ehrlich ascites assay^46^, while quercetin-4′-O-glucoside proved its anticancer potential against hepatoblastoma cell line (HepG-2), PC3 and colorectal adenocarcinoma (HT-29) cell lines^47^. Importantly, esculin (Fig. 13) showed a high binding affinity with a score of -6.210 despite being smaller in size than the previous flavonoid glycosides. It formed three H-bonds as a H-bond donor, one H-bond as a H-bond acceptor, and one H-π interaction. Esculin was reported to have good anticancer activity via induction of apoptosis and autophagy in human glioblastoma multiforme cells (T98G) and human anaplastic astrocytoma cells^48^. Rosmarinic acid (Fig. 14) came very close to esculin with a score of − 6.144 through the formation of two H-bonds as a H-bond donor, one as a H-bond acceptor, and one as H-π interaction. Rosmarinic acid showed anticancer activity against many types of cancer as colon, skin, lung, and ovarian cancers^49^. Finally, came chlorogenic acid (Fig. 15) and the triterpene alpha-amyrin (Fig. 16) with scores of − 5.616 and − 5.272, respectively. Chlorogenic acid proved its activity against breast carcinoma^50^, while alpha-amyrin was reported to have anticancer activity against hepatocellular carcinoma via an in-silico docking study^51^. This result may explain the promising synergistic anticancer activity of the different fractions of *L. hispidulus* optimized extract against prostate carcinoma cell lines.

**Table 6:** Docking results with Hexokinase-2 enzyme.

	S (kcal/mol)	Amino acids	Interacting groups	Type of interaction	Length
Native ligand	− 8.018	Asp657	OH	H-bond donor	2.60
		Asp657	CH_2_	Non-classical H-bond	3.48
		Asn656	OH	H-bond acceptor	3.02
		Asn656	OH	H-bond acceptor	3.28
		Glu708	NH	H-bond donor	2.69
		Glu708	OH	H-bond donor	2.75
		Asn683	OH	H-bond acceptor	2.88
		Gly681	SO_2_	H-bond acceptor	2.88
		Thr680	SO_2_	H-bond acceptor	2.84
		Lys621	CN	H-bond acceptor	2.63
		Lys738	Phenyl	cation-Pi interaction	4.38
Alpha-amyrin	− 5.272	Asn656	OH	H-bond acceptor	3.33
		Glu708	OH	H-bond donor	2.81
Apigenin-7-O-glucoside	− 7.412	Asp657	OH	H-bond donor	2.98
		Glu742	OH	H-bond donor	3.44
		Lys621	O	H-bond acceptor	3.89
Chlorogenic acid	− 5.616	Asp657	OH	H-bond donor	3.15
		Asn683	OH	H-bond acceptor	3.06
		Glu708	OH	H-bond donor	3.42
		Cys606	Phenyl	H-Pi interaction	4.50
		Pro605	Phenyl	H-Pi interaction	4.46
Esculin	− 6.210	Asp657	OH	H-bond donor	2.95
		Asn656	OH	H-bond acceptor	2.79
		Glu708	OH	H-bond donor	2.93
		Cys606	OH	H-bond donor	2.95
		Ser603	Phenyl	H-Pi interaction	3.89
Kaempferol-3-O-glucuronide	− 6.858	Asp657	OH	H-bond donor	3.10
		Glu708	OH	H-bond donor	2.89
		Pro605	Pyran	H-Pi interaction	4.31
		Pro605	Phenyl	H-Pi interaction	4.63
Luteolin-7-O-glucoside	− 7.342	Asp657	OH	H-bond donor	3.03
		Asn656	OH	H-bond acceptor	2.90
		Glu708	OH	H-bond donor	3.27
		Cys606	Pyran	H-Pi interaction	4.34
		Pro605	Pyran	H-Pi interaction	3.90
Quercetin-4′-O-glucoside	− 6.289	Asp657	OH	H-bond donor	2.82
		Asn656	OH	H-bond acceptor	2.83
		Glu708	OH	H-bond donor	2.80
		Ser603	Phenyl	H-Pi interaction	3.84
		Gln608	OH	H-bond donor	2.94
		Ser614	OH	H-bond acceptor	3.06
Rosmarinic acid	− 6.144	Asp657	OH	H-bond donor	2.86
		Glu708	OH	H-bond donor	2.81
		Lys621	O	H-bond acceptor	2.95
		Pro605	Phenyl	H-Pi interaction	4.30

S (kcal/mol), docking score in kilocalorie per mole; Asn, Asparagine; Asp, Aspartic acid; Cys, Cysteine; Glc, Glucose; Gln, Glutamine; Glu, Glutamic acid; Gly, Glycine; Lys, Lysine; Pro, Proline; Ser, Serine; Thr, Threonine.

## Conclusion

The comprehensive profiling of *L. hispidulus* optimized ethanolic extract revealed the presence of thirty-six phenolic compounds which were identified for the first time in this plant species using LC-qTOF-MS. These compounds include the glycosides of (luteolin, quercetin, kaempferol, apigenin, isorhamnetin, and daidzein), coumarines (esculin, esculetin and daphnetin), phenolic acids (chlorogenic, caffeic, quinic, *P*-coumaric and rosmarinic). GC–MS/MS also was performed for the first time revealing the presence of eighteen fatty acids and hydrocarbons as palmitic acid, myristic acid, α-linolenic acid, hexadecanoic acid, oleic acid, behenic acid, and triterpenes (α- and β-amyrin). Methylene chloride and ethyl acetate were the most active fractions against the PC3 cell line. They showed very close IC_50_ values (19 and 19.6), respectively. It is worthy to mention, that all the successive fractions (except for the butanol fraction) showed a significantly higher anticancer activity against the PC3 cell line than doxorubicin at the highest concentration (50.00 µg/ml). Apigenin-7-*O*-glucoside showed the highest inhibitory effect of HEK-2 enzyme with a binding score of − 7.412, followed by luteolin-7-*O*-glucoside, kaempferol-3-*O*-glucuronide, quercetin-4ˋ-*O*-glucoside, esculin, rosmarinic acid, chlorogenic acid and finally alpha-amyrin. The results of the docking study showed consistency with the results of the anticancer activity study on the successive fractions, as the synergistic activity could be due to the flavonoid glycosides, coumarins, phenolic acids, and triterpenes combined. This study could be a starting point for many future studies on *L. hispidulus* to further investigate its biological potential.

## Data Availability

The datasets used and/or analyzed during the current study are available from the corresponding author upon reasonable request.

## Abbreviations

ABTS: 2,2′-Azino-bis(3-ethylbenzothiazoline-6-sulfonic acid)
Asn: Asparagine
Asp: Aspartic acid
CDS: Calibration delivery system
Cys: Cysteine
DI-water: Deionized water
EDTA: Ethylenediaminetetraacetic acid
EI: Electron ionization
ESI: Electron spray ionization
EV: Electronvolt
GC/MS: Gas chromatography-mass spectrometry
Glc: Glucose
Gln: Glutamine
Glu: Glutamic acid
Gly: Glycine
HEK-2: Hexokinase-2 enzyme
5HFU: Crystal structure of Human Hexokinase-2
HELA: Human Cervical carcinoma cellline
HepG-2: Hepatoblastoma cell line
HPLC: High-performance liquid chromatography
HT-29: Colorectal adenocarcinoma cell line
IC_50_: Half-maximal inhibitory concentration
Kcal/mol: Kilocalorie per mole
KV: Kilovolt
LC-qTOF-MS: Quadrupole Time-of-Flight Liquid chromatography-mass spectrometry
Lautumnalis: Leontodon autumnalis
*L*. *filli*: *Leontodon* *filli*
*L*. *hispidus*: *Leontodon hispidus*
*L*. *hispidulus*: *Leontodon* *hispidulus*
*L.**saxatilis*: *Leontodon* *saxatilis*
Lys: Lysine
MOE: Molecular operating environment
MONA: Mass Bank of North America
MP-WS: Mobile phase working solution
NIST: National Institute of Standards and Technology
OPM2: Plasma cell myeloma cell lines
PC3: Human Prostate carcinoma cell line
PDB: Protein data bank
Pro: Proline
Rham: Rhamnose
RESPECT: Database for phytochemicals
RMSD: Root mean square deviation
Ser: Serine
SRB: Sulforhodamine-B assay
T98G: Human glioblastoma multiforme cells
Thr: Threonine
TIC: Total Ion Chromatogram
TMCS: Trimethylchlorosilane
TROLOX: 6-Hydroxy-2,5,7,8-tetramethyl chroman-2-carboxylic acid
UPLC: Ultra-performance liquid chromatography
